# Recombinant M2e Protein-Based ELISA: A Novel and Inexpensive Approach for Differentiating Avian Influenza Infected Chickens from Vaccinated Ones

**DOI:** 10.1371/journal.pone.0056801

**Published:** 2013-02-21

**Authors:** Farhid Hemmatzadeh, Sumarningsih Sumarningsih, Simson Tarigan, Risa Indriani, N. L. P. Indi Dharmayanti, Esmaeil Ebrahimie, Jagoda Igniatovic

**Affiliations:** 1 School of Animal and Veterinary Sciences, The University of Adelaide, Adelaide, Australia; 2 School of Veterinary Science, The University of Melbourne, Melbourne, Australia; 3 Indonesian Research Centre for Veterinary Science, Bogor, Indonesia; 4 Research Centre for Infectious Diseases, School of Molecular and Biomedical Science, The University of Adelaide, Adelaide, Australia; The University of Hong Kong, China

## Abstract

Available avian influenza (AIV) serological diagnostic tests cannot distinguish vaccinated from naturally infected birds. Differentiation of vaccinated from infected animals (DIVA) is currently advocated as a means of achieving the full control of H5N1. In this study, for the first time, recombinant ectodomain of M2 protein (M2e) of avian influenza virus (H5N1 strain) was used for the DIVA serology test. M2e was cloned into pMAL-P4X vector and expressed in *E. coli* cells. We used Western blot to recognize the expressed M2e-MBP protein by chicken antisera produced against live H5N1 virus. Also, the specificity of M2e-MBP protein was compared to the M2e synthetic peptide via ELISA. In M2e-MBP ELISA, all sera raised against the live avian influenza viruses were positive for M2e antibodies, whereas sera from killed virus vaccination were negative. Furthermore, M2e-MBP ELISA of the field sera obtained from vaccinated and non-vaccinated chickens showed negative results, while challenged vaccinated chickens demonstrated strong positive reactions. H5N1-originated recombinant M2e protein induced broad-spectrum response and successfully reacted with antibodies against other AIV strains such as H5N2, H9N2, H7N7, and H11N6. The application of the recombinant protein instead of synthetic peptide has the advantages of continues access to an inexpensive reagent for performing a large scale screening. Moreover, recombinant proteins provide the possibility of testing the DIVA results with an additional technique such a Western blotting which is not possible in the case of synthetic proteins. All together, the results of the present investigation show that recombinant M2e-MBP can be used as a robust and inexpensive solution for DIVA test.

## Introduction

Highly pathogenic avian influenza virus (AIV) of H5N1 subtype has become endemic in poultry in some countries, especially in Southeast Asian Countries [1]. The continuous presence of H5N1 in the environment has considerable veterinary and social consequences. Due to devastating losses of H5N1 in most poultry, life-long vaccination of commercial poultry has become a necessity [2], [3], [4]. H5N1 is also a zoonotic agent which has caused human death in number of countries through direct contact of human with infected poultry [5], [6]. Therefore, in the context of both veterinary and social standpoints, it is important to reduce the level of H5N1 in the environment [7].

Multiple vaccinations are expensive and in many instances not entirely effective enabling H5N1 to persist in the environment and mutate through the process known as “antigenic drift” [4], [8]. Surveillance of vaccinated poultry for H5N1 that is, differentiation of vaccinated from infected animals (DIVA), is advocated as a mean to achieve the full control of H5N1, leading to eventual eradication [9], [10]. The main issue is that the common available diagnostic tests can not differentiate vaccinated from naturally infected birds.

To overcome this limitation, several DIVA strategies have been attempted; the most feasible approach is the use of subunit-based strategy which targets differential rate of propagated avian influenza proteins between killed virus (vaccine) and naturally infected birds. Hemagglutinin (H) is the most used target subunits [11]. HA allows serologic surveillance in both infected and vaccinated birds. However, the major drawback of HA-based strategy is the high number of HA molecules per virion (500) giving positive result in infected and vaccinated birds. In addition, influenza virus evades humoral immune response by rapid mutation of HA and NA coat proteins (HA and NA) [12].

A major improvement was the use of nonstructural protein 1 (NS1) as the target subunit which has zero copy number per mature virion [13]. Infected host cells contain large quantities of this protein, but NS1 does not package in virion [13]. As a result, a DIVA test based on differential antibody response to NS1 protein can differentiate infected from vaccinated birds [11]. However, it has been shown that the accuracy of NS1-based DIVA test decreases by the time and produces non-specific reactions [13].

Another surface segment of influenza virus, the matrix protein 2 (M2), is a transmembrane integral protein where it exists as homotetramer, each monomer contains 96 amino acid with 3 domains: a small external domain (M2e) comprising 23 amino acids, a transmembrane domain (19 amino acids) and a cytoplasmic domain (54 amino acid) [14], [15].

The matrix protein 2 (M2) is receiving increased attention since unlike HA and NA, the extracellular domain of the M2 protein (M2e) is not subjected to severe immune selection pressure and is very well conserved [12]. Vaccinated mice with M2e protein showed complete protection against challenges with highly pathogenic (homologous and heterologous) human influenza [16], [17]. Indeed, M2 protein revealed a high potential as a vaccine for prevention of swine influenza virus disease [18]. In spite of its high potential, there is no report on application of M2e as vaccine in poultry.

The extracellular domain of the M2 protein (M2e protein) is abundantly expressed on the surface of infected cells, while it is present in small quantities in the mature virions (20–60 molecules per vision) [19], [20]. Hence, humans/animals vaccinated with conventional inactivated influenza vaccines are not expected to have M2e-binding antibodies [21].

Only a limited number of studies have examined M2e as a diagnostic marker in DIVA test. Lambrecht, et al. (2007) and Kim et al (2010) utilized the M2e synthetic peptide in ELISA and were successful in discrimination of infected and vaccinated birds indicating the potential of M2e for DIVA test [22], [23]. This approach (synthetic peptide-based ELISA) requires a large amount of synthetic peptide, which is expensive for routine flock monitoring [24] and restricts its application. Another shortcoming of the synthetic peptide is that the result of ELISA in doubtful samples cannot be examined by another serological method such as Western blotting. The mentioned limitations might be overcome by production of M2e recombinant protein in a highly efficient platform such as *E.coli* and utilizing DIVA test based on the recombinant protein.

In the present study, the M2e from a H5N1 Indonesian strain expressed in *E. coli* as a recombinant protein and evaluated by ELISA for its ability to detect antibodies to M2e antigen in reference antisera and in chicks infected or vaccinated with different subtypes of AIV. This study provides an efficient and affordable method for differentiation of vaccinated from naturally infected chicken which is an industrial demand.

## Materials and Methods

### Synthesis of the Me2 Gene Construct

We selected consensus open reading frame of the M2e domain of M2 protein based on the multiple alignment of available H5N1 sequences of H5N1 Indonesian strains in GeneBank (http://www.ncbi.nlm.nih.gov/). Selected sequence had the first 72 nucleotides of M2 mRNA of A/Indonesia/CDC540/2006 strain (accession number EU014132.1). The obtained sequence was optimized for the expression in *E. coli* since the wild-type M2e gene contained rare codons with a considerable frequency and several negatively cis-acting motifs, which might hamper its expression in *E. coli*. The optimised gene was synthesized, cloned into NT1 cloning vector with *Bam* HI and *Sal* I restriction sites at the 5′ and 3′ ends of the gene, respectively. The correct orientation of the M2e open reading frame was confirmed by sequencing (GENEART AG; Gewerbpark- Regensburg, Germany; www.geneart.com).

### Cloning and Expression of the M2e Recombinant Protein

M2-NT1 vector was transformed into BL21 strain of *E. coli* according to Sambrook and Russell protocol [25]. After amplification, the plasmid was isolated, then digested using restriction enzymes *Bam* HI and *Sal* I (New England Biolabs. Inc, Ipswich, MA, USA), and ligated into pMAL-P4X expression vector (New England Biolabs. Inc, Ipswich, MA, USA). This expression vector carried maltose binding protein (MBP) as a fusion protein for further purification. Finally, the recombinant vector of M2e-pMAL was constructed. The M2e-pMAL expression vector was transformed into *E. coli*, BL21 cells using electroporation method. Then, the grown colonies were verified to contain M2e-pMAL by PCR and sequencing in both directions.

For expression of recombinant M2e-MBP protein, a single colony of transformed BL21 cells was cultured in a rich Luria-Bertani Medium (LB) (10 g Tryptone, 10 g NaCl, 5 g yeast extract and 0.1% D-glucose per liter of distilled water) containing 30 µg/ml of filtered sterile Ampicillin. The 10 ml overnight culture was transferred in 1 L of LB medium containing Ampicillin and incubated at 37°C until optical density at 600 nm (OD_600_) reached to about 0.5, then the production of M2e-pMAL protein was induced by application of 0.3 mM isopropyl β-D-1-thiogalactopyranoside (IPTG) (Sigma, St Louis, MO, USA) with subsequent incubation for 3 h at 250 rpm at 37°C. A non-induced culture was used as a negative control. The culture fluid was centrifuged at 5,000×g for 20 min at 4°C in (Sorvall RC 5B centrifuge). The supernatant was discarded, and the cells were resuspended in 400 ml Tris/sucrose buffer (30 mM Tris-HCl, 20% sucrose, 1 mM EDTA, pH 8.0). Then, the suspension incubated for 5–10 minutes at room temperature with shaking, or stirring, and centrifuged at 8000×g at 4°C for 10 minutes. The pelleted cells were resuspended in 400 ml ice-cold 5 mM MgSO4, incubated for 10 minutes in an ice-water bath and centrifuged as mentioned above. The supernatant was termed “cold osmotic shock fluid”.

### Purification of M2e-MBP Fusion Recombinant Protein

The “cold osmotic shock fluid” containing M2e-MBP loaded onto a column of amylose affinity resin (New England Biolabs, Beverly, Mass., USA), washed overnight at 4°C with 5 volumes of column buffer (20 mM Tris-HCl, 200 mM NaCl, 1 mM EDTA, 1 mM NaN_3_ and 1 mM Dithiothreitol). Then, the M2e-MBP protein eluted with column buffer containing 10 mM maltose (Sigma, St Louis, MO, USA). The first 10 fractions were collected and protein concentration of each fraction monitored by measurement of OD_280_ on a NanoDrop system (Thermo Scientific, DE, USA). The fractions that contained measurable protein were desalted and concentrated by ultrafiltration in Vivaspin size exclusion centrifugal membrane tube with cut-off 30,000 Dalton (Sartorius Stedim Biotech, Goettingen Germany). Protein concentration of the purified protein was measured using NanoDrop, adjusted at 800 µg/ml and stored in aliquots at −70°C.

### Analysing of Purified Protein by SDS-PAGE

Purified protein was analysed by *sodium dodecyl sulfate polyacrylamide gel* electrophoresis (SDS-PAGE) using gels with 12.5% concentration of acrylamide. Pre-stained molecular weight markers (New England Biolabs. Inc, Ipswich, MA, USA) and purified MBP protein (previously purified) were included as standards.

### Western Blotting

Purified recombinant M2e-MBP protein was ran on 12.5% SDS-PAGE and transferred to a nitrocellulose membrane. Molecular weight markers (New England Biolabs, Beverly, Mass, USA) were also included. After transferring, the membrane was blocked using 10% bovine serum albumen (BSA) in PBS containing 0.5% Tween 20 for 2 h at room temperature. Test sera (Table 1) were diluted 1∶500 in dilution buffer (PBS- Tween containing 1% BSA) and incubated for 1 h. The membrane was probed for 1 h at room temperature with a rabbit anti-chicken IgG conjugated to horse-radish peroxidase (Millipore, Temecula, California, USA). After washing with PBST, protein bands were visualized using diaminobenzidine (DAB) (Sigma-Aldrich Pty. Ltd) as substrate.

**Table 1:** Reference (Avian Influenza Virus) AIV antisera used in the study.

	Avian influenza strain used for immunization	Type	HI Titre log_2_ 1
1	A/Chicken/Scotland/1959	H5N1	7^2^
2	A/Ostrich/Denmark/72420/1996	H5N2	7
3	A/Turkey/Wisconsin/1/1966	H9N2	9
4	A/Tky/England/96	H3N2	7
5	A/Duck/Alberta/35/76	H1N1	9
6	A/African starling/England-Q/983/79	H7N1	8
7	A/Duck/England/1/1956	H11N6	9
8	A/Duck/Ukraine/1/1963	H3N8	6
9	A/Duck/Germany/1215/1973	H2N3	6
10	A/Turkey/England/647/77	H7N7	7
11	A/Ck/Viet Nam/8/2004	H5N1	ND^3^
12	A/Chicken/Konawi Selatan/8/2004 H5N1	H5N1	ND^3^

1 Homologous HI Titre.

2 Source of challenged and vaccinated antibodies.

3 ND: HI Titre not detected.

### Synthetic Peptides

Two synthetic peptides corresponding to the M2e recombinant sequences were made; the M2e-23 peptide, corresponding to amino acid position 2 to 24, with the sequence SLLTEVETPTRNEWECKCSDSSD, and the M2e-18 peptide, amino acid position 2 to 18, with the sequence SLLTEVETPTRNEWECKC. Both peptides were synthesized by PEPTIDE 2.0 (Chantilly, VA, USA) with a minimum of 85% purity as measured by high performance liquid chromatography. Both peptides had high solubility in distilled water.

### Reference AIV Antisera

Antisera against different strains and subtypes of AIV were obtained from the Veterinary Laboratory Agency (New Haw, Addlestone, UK) and CSIRO Australian Animal Health Laboratory, Geelong, Victoria (Table 1). All sera were produced in specific pathogen-free (SPF) chicks by inoculation of either inactivated AIV only (as a source of vaccine antibodies), or by inoculation of inactivated AIV followed by challenge with live AIV of the same H and N subtype two weeks after each immunization (as a source of challenged antibodies). In all of the cases, the SPF chicks were immunized twice with the inactivated virus and then inoculated with live virus only in challenged groups. Haemagglutination inhibition (HI) titers were determined in each serum by the suppliers using the homologues antigen similar to the one used for immunization.

### Optimization of Recombinant M2e-MBP ELISA

A checkerboard titration of the M2e-MBP antigen and AIV positive and negative sera were initially performed to determine the optimal OD at 450 nm (OD_450_) of each positive and negative serum. Briefly, purified M2e-MBP protein, diluted in 0.1 M carbonate– bicarbonate buffer (pH 9.6), at concentrations of 200 to 0.82 µg/ml, was used to coat the 96-well flat bottom microtitre plate (*Sarstedt*, Nümbrecht, Germany). The coated plates were incubated at room temperature (RT) overnight and then washed three times with high salt wash buffer (WB), (NaCl, 37.5 g, KCl, 0.2 g, Na2HPO4, 1.15 g, KH2PO4, 0.5 g, Tween 20, 0.5 ml in 1 L distilled water, pH 7.2). The unsaturated sites were blocked by 5% BSA in PBS (200 µl/well) at RT for 2 h, and subsequently washed three times with WB. Test sera were diluted 1∶25 to 1∶800 in dilution buffer (DB) (0.1 M Tris pH 7.4, 0.5 M NaCl, 1 mM Na2EDTA, 2% w/v BSA, 3% w/v Triton X-100, 3% w/v Tween 20) and added (100 µl/well) in duplicate to the wells and incubated at RT for 1 h. Positive and negative controls were included in all assays in quadruplicate. The plates were washed three times with PBS-T. Horseradish peroxidise labelled rabbit anti-chicken IgG (Millipore, Temecula, California, USA), 100 µl/well were added and incubated for 1 h at RT. After washing, the substrate solution (100 µg/ml of tetramethylbenzidine substrate (TMB) (Sigma, St Louis, MO, USA) in citrate buffer (pH 8), containing hydrogen peroxide (100 µl of 0.6% H_2_O_2_), was added (100 µl/well) and the plates were incubated for 10–15 min. The colour reaction was stopped by adding 1 M sulphuric acid, and OD_450_ was determined.

### Optimization of Synthetic Peptide ELISA

Both M2e-23 and M2e-18 synthetic peptides were used in ELISA as coating antigens. Briefly, 1 mg of each lyophilized peptide was dissolved in 1 ml of sterile distilled water, and 2-fold serial dilutions of 160 to 0.31 µg/ml of the peptide made in 0.1 M carbonate–bicarbonate buffer, pH 9.6. Diluted peptides were used to coat the 96-well flat bottom microtiter plates (Maxisorp, NUNC). All steps, including the checkerboard titration, were performed as described for M2e-MBP ELISA.

In both M2e peptides and M2e-MBP ELISAs, for each serum sample, 4 wells were used: 2 wells were coated by the antigen whereas 2 wells were not coated with any antigen and served as an internal control for each serum. For each serum, the mean OD_450_ of antigen negative wells subtracted from the mean OD_450_ of antigen positive wells and the obtained OD_450_ was termed as “corrected OD_450_ value”. A serum was considered positive when its corrected OD_450_ value was greater than the mean corrected OD_450_ for negative sera plus 2 times standard deviation (cut-off value).

### Challenged Experiment: Simulation the Efficiency of the Presented Recombinant M2e-based ELISA Method in Comparison to the Common HA-based Method (HI Titre) for DIVA Test

The aim of this experiment was to simulate the condition and measure the sensitivity of the presented recombinant M2e-based ELISA method when the vaccinated chickens infect with the live virus. In addition, the efficiency of our method was compared with the HI titre (common HA-based method).

Chicken sera resulting from an experimental vaccination/challenge experiment were provided by the Indonesian Research Centre for Veterinary Science, Bogor. Twenty chicks, 21 days old, were sourced from non-vaccinated AI free broiler farm and divided into 2 groups, each with 10 chicks. Chickens in the first group were vaccinated with a single dose of an experimental inactivated vaccine prepared from A/Ck/West Java/Pwt-Wij/2006 strain of H5N1 (Accession No; EU124148). Chickens in the second group were remained unvaccinated and kept in the same condition but in a separate isolation units.

Three weeks after vaccination, all birds were tested for HI antibody titres using the homologous HA antigen (A/Ck/West Java/Pwt-Wij). Then, chicks in both groups were transferred to isolation units, housed within the PC3 facility. After that, chicks in the first group were challenged with 10^6^ ELD_50_ in 0.1 ml via intranasal inoculation with A/Ck/West Java/Pwt-Wij/2006. Chicks in the second group were remained unvaccinated and unchallenged. Two weeks after challenge, sera were collected from all chicks in both groups and tested individually for HI antibody titres using the HA antigen A/Ck/West Java/Pwt-Wij and also M2e ELISA, to compare the ability of these methods in monitoring the infection of vaccinated chickens. In addition, in first group, the responses of M2e ELISA and HI Titre methods to challenged infection were compared by T-test statistics using Minitab 16 package (www.minitab.com).

### Large Scale DIVA Test Using the Recombinant M2e-based ELISA

The goal of this experiment was to evaluate the robustness of the recombinant M2e ELISA method in the field scale. Three test groups were examined, including (a) Negative group (non-infected and non-vaccinated): 204 field sera from commercial broiler and layer flocks in Australia and Indonesia which were confirmed to be AIV antibody free by an IDEXX AIV antibody test (IDEXX Laboratories, Inc) and were obtained from the diagnostic laboratory, School of Veterinary Science, the University of Melbourne, (b) Vaccinated group, sera (334 in total) were collected from vaccinated commercial broiler and layer flocks in Indonesia, and (c) Infected group: 56 sera were collected from infected chickens from different farms in Indonesia.

Corrected ODs (450 nm) of each serum in recombinant M2e ELISA were recorded individually and compared between different test groups. Furthermore, analysis of variance mean comparisons by Tukey test was performed to evaluate the ability of M2e-based ELISA in distinguishing the infected sera from the vaccinated and non-vaccinated sera (Table 2).

**Table 2:** Mean comparisons and analysis of variance of recombinant M2e-MBP ELISA results on sera from challenged, negative, and vaccinated tested groups.

Mean comparison					
Test group		Mean	Standard Deviation	Mean comparison by Tukey method at 99% Confidence level^1^	
Infected		0.9269	0.2711	A	
Vaccinated		0.2124	0.1981	B	
Negative		0.1955	0.0108	B	
**Analysis of Variance**					
**Source of variation**	**Degree of freedom**		**Mean squares**	**F-value**	**P-value** 2
Test group	2		13.197	355.44	0.0001
Error	591		0.037		

1 Means that do not share a letter are significantly different.

2 Highly significant.

### ELISA Cut-off Values

The cut-off values for the ELISAs were calculated from the results of sera of H5N1 infected (standard and challenge experiment groups) and vaccinated chickens (field and challenge experiment groups) using the two-graph receiver operating characteristic (TG-ROC) analysis [26], [27].

### Ethics Statement

All animal experiments were performed at the Indonesian Research Centre for Veterinary Science, Bogor, Indonesia. The study proposal was approved by the institutional Research Committee.

The animals were managed by a veterinarian who specializes in animal studies based on the guidelines of the National Health and Medical Research Council of Australia. All birds were bleed via brachial vein during the experiment and cardiac puncture at the terminal step just after CO2 euthanization.

## Results

### M2e Consensus Sequence Used for Expression

In total, 64 nucleotide sequences of the M2e gene of deposited Indonesian H5N1 strains in GeneBank (with human and avian origin) were aligned. The obtained consensus M2e sequence from this alignment was that of A/Indonesia/CDC540/2006 H5N1 strain since it shared the highest identity matrix, >95%, with other Indonesian H5N1 strains. The length of sequence was 72 nucleotides and also had the highest hydrophilicity profile among the compared M2e amino acid sequences (result not shown). The hydrophilicity is one of the factors that influence both antigenicity and antibody binding avidity. High hydrophilicity increases the chance of detection of the specific antibodies. We optimized the codons of this 72 bp long M2e sequence, cloned and expressed in *E. coli*, BL21 cells.

### Characterization of the Recombinant M2e-MBP Protein

**Figure 1:**
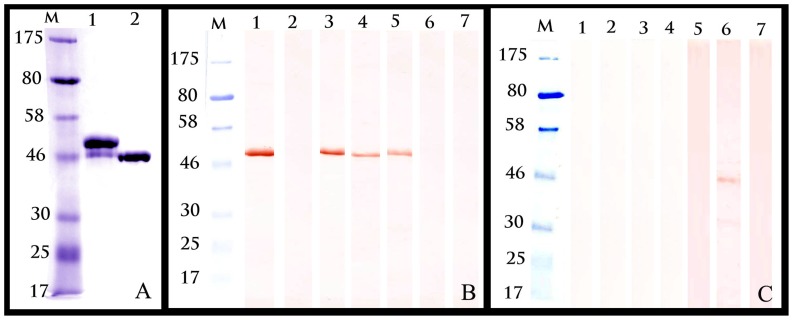
SDS-PAGE and Western blot results of M2e-MBP recombinant protein. A) Purified recombinant M2e-MBP (lane 1) and MBP (lane 2) proteins and pre-stained molecular marker (M) analysed by SDS–PAGE (12.5%) and stained using Coomassie Brilliant Blue. (B & C) Western blot analysis of purified M2e-MBP (1B) and MBP (1C) with reference AIV antisera to: (1) live A/Chicken/Scotland/1959 (H5N1); (2) inactivated H5N1; (3) live A/Ck/Viet Nam/8/2004 (H5N1); (4) live A/Turkey/Wisconsin/1/1966 (H9N2); (5) live A/Ostrich/Denmark/72420/1996 (H5N2); (6)sera from commercial non-vaccinated non-infected; (7) SPF chicken sera.

Maximum expression level of M2e-MBP was detected at 3 h after induction. SDS-PAGE analysis of the purified M2e-MBP protein demonstrated the presence of two 45.1 and 42.5 kd bands (Figure 1A). The 45.1 kd band corresponded to the expected recombinant protein containing 2.6 kd M2e and 42.5 kd MBP, whereas 42.5 kd band corresponded to the MBP alone. Therefore it is likely to be truncated M2e-MBP protein.

### Confirmation of the Expressed Recombinant Protein with Western Blot

The purified M2e-MBP fusion protein reacted in Western blot with AIV chicken sera that included antisera to live H5N1 (A/Chicken/Scotland/1959 and A/Ck/Viet Nam/8/2004), live H5N2 (A/Ostrich/Denmark/72420/1996), and live H9N2 (A/Turkey/Wisconsin/1/1966) (Figure 1B). H5N1, as well as H5N2 and H9N9 antisera reacted with 45.1 kd band which contains M2e. None of these sera reacted with MBP alone, indicating that the recognition of 45.1 kd band is due to the presence of M2e and not MBP (Figure 1B). Antisera to inactivated H5N1 (vaccine) did not bind to the M2e-MBP indicating that immunisation with inactivated virus did not give rise to the M2e antibodies, documenting the robustness of the presented method.

Pool of field sera from AIV free, non-vaccinated chicks, did not react with the M2e-MBP protein in Western blot (Figure 1B). However, a weak reaction was observed with alone MBP protein (Figure 1C, lane 6), likely due to the higher concentration of MBP when MBP used alone in Western Blotting.

### Optimization of Recombinant M2e-MBP and Synthetic M2e Peptide ELISA

**Figure 2:**
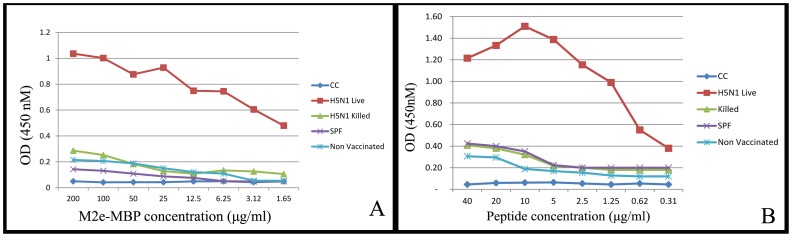
Optimization of recombinant M2e-MBP protein (A) and M2e-18 synthetic peptide (B) as coating antigens in ELISA. Anti-H5N1 live, anti-H5N1 killed, SPF and sera from non-vaccinated commercial chickens (field sera) were diluted 1∶100 and incubated with M2e-MBP or M2e-18 used for coating at various concentration. Reactivity of sera with coating antigen was detected by rabbit anti-chicken IgG-HRP. Absorbance (OD) of conjugate control (CC) was measured for each plate.

Since Western blot analysis confirmed that the recombinant M2e-MBP fusion protein is antigenic and can differentiate live infected from vaccinated antisera, feasibility of using this antigen in ELISA was assessed. In addition, the synthetic M2e-18 peptide ELISA was used for comparison as was previously shown to be able to discriminate between infected and vaccinated chicks [22], [28]. Initially, both peptide and M2e-MBP ELISA were optimized for the amount of coating antigen and dilution of antisera. For M2e-MBP as coating antigen and antisera to live H5N1, the lowest background and maximum absorbance were determined to be at approximately 25 µg/ml for M2e-MBP antigen and 10 µg/ml for M2e-18 peptide (Figure 2). The highest ratios of OD_450_ for H5N1 live and killed antisera, with minimum non-specific reaction, was obtained at sera dilution of 1∶100 and consequently this dilution was used for all test sera in ELISA.

### Comparison of Recombinant M2e-MBP and Synthetic M2e Peptide ELISA in Detection of Live AIV Exposure

**Figure 3:**
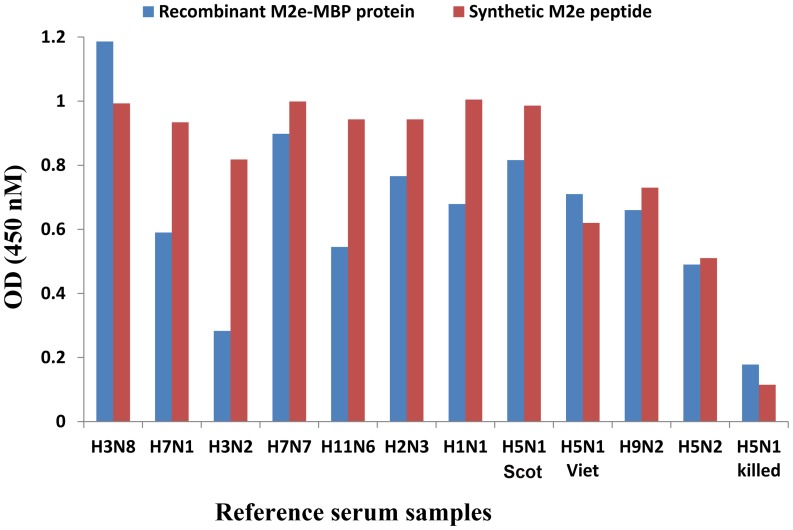
Comparison of recombinant M2e-MBP protein and synthetic M2e peptide ELISA for detection of M2e antibodies in reference sera. All sera were generated against live AIV of indicated subtypes, except for H5N2 killed virus.

Reactivity of sera from chicks immunized with 11 different subtypes of AIV, with HI titres between 6 to 9 (log 2 base) were compared in recombinant M2e-MBP and synthetic M2e peptide ELISA (Figure 3). As it can be inferred from Figure 3, a considerable agreement exists between the recombinant M2e-MBP and synthetic M2e peptide ELISA for all sera. The highest difference was observed for H3N2 antiserum, where its reaction with the M2e-MBP protein was lower in comparison to its reaction with the M2e synthetic peptide. One of the possible reasons of this difference (in the case of H3N2) is the lower signal to noise ratio for M2e-MBP ELISA comparing to synthetic peptide ELISA. It should be noted that higher signal of synthetic peptide ELISA is related to the higher purity of synthetic peptide.

As presented in Figure 3, two live H5N1 strains, Scotland and Vietnam, strongly reacted with recombinant M2e-MBP and synthetic M2e peptide, whereas sera to inactivated H5N1 did not react. This clearly demonstrates the high performance of M2e in DIVA test for distinguishing vaccinated from infected chickens. Significant positive Pearson correlation (p = 0.05, 67%) was observed between M2e-MBP and synthetic M2e peptide which highlights the possibility of employing inexpensive recombinant M2e-MBP instead of synthetic M2e protein.

Of interest was that antisera to live AIV of subtypes other than H5N1, including H11N6, H7N7, H1N1, H7N1, H2N3, H9N2, H3N8 and H5N2, all reacted in ELISA with both M2e-MBP and M2e peptide while M2e sequence was derived from H5N1 strain. In line with this finding, De Filette et al., (2008) showed that vaccines based on M2e can induce broad-spectrum immunity against influenza in mice and is the best candidate for M2e-based universal influenza vaccine [16]. Similarly, broad protection against different H1 swine influenza virus was achieved by application of swine influenza M2 protein [18].

### High Sensitivity of Recombinant M2e-MBP ELISA for DVIA Test, Superiority Over the Common HA-based Method (HI Titre)

**Figure 4:**
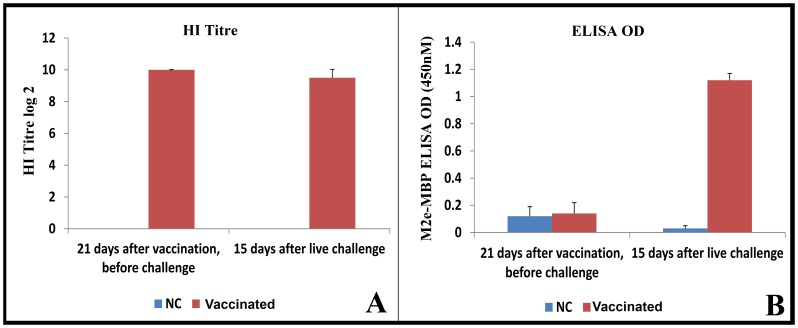
Comparison of HI titres and M2e-MBP ELISA OD in chicks vaccinated and then challenged by A/Ck/West Java/Pwt-Wij/2006.

Figure 4 compares the response of HI titre and recombinant M2e ELISA in monitoring the infection in vaccinated chickens with live virus. HI titre could not distinguish the further infection of vaccinated chicken with live virus (A/Ck/West Java/Pwt-Wij/2006) (Figure 4A). In contrast, while vaccinated and non-vaccinated chickens have the similar ELISA OD before infection, the level of ELISA OD sharply increased by more than 8 times (from 0.14 to 1.2) (Figure 4B). The cut-off point for M2e-MBP ELISA was 0.58. T-test statistically confirmed the highly significant response of M2e ELISA test to live virus infection at p = 0.00001(data not shown). This result confirms high efficiency of the presented recombinant M2e-based ELISA method in DIVA test and its superiority over the common HA-based method.

### Evaluation of M2e-MBP and M2e Peptide ELISA with Field Serum Samples (Large Scale DIVA Test)

**Figure 5:**
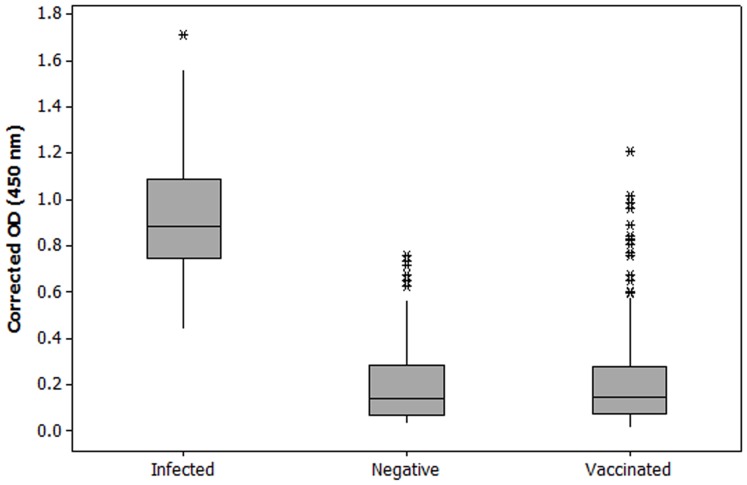
Large scale (field) application of recombinant M2e-MBP ELISA results on sera from infected (challenged), non-vaccinated, and vaccinated groups. Cut-off value (0.58) calculated as mean corrected OD for negative samples plus 2 standard deviations (0.364).

Specificity of M2e-MBP and M2e peptide ELISA were also evaluated using chicken sera from non-vaccinated and vaccinated commercial layers and non-vaccinated broilers as described in materials and methods. Sera from non-vaccinated layers were negative in both M2e-MBP and M2e peptide ELISA, except for 6 sera that were positive in M2e-MBP ELISA with the OD_450_ value greater than 0.58 (false positive rate  = 2.9%) (Figure 5). Western blot analysis of these sera revealed that they had reacted with the MBP contained in the fusion protein and but not against M2e (data not shown). In vaccinated group, 19 out of 334 were also positive in M2e-MBP ELISA (false positive is 5.6%) (Figure 5).

As it can be inferred from Figure 5, the presented M2e-MBP ELISA is highly efficient in differentiation of infected from non-infected (negative) and vaccinated groups in field level (large scale). Analysis of variance revealed a highly significant difference between infected group in comparison to non-infected and vaccinated groups with respect to ELISA OD values (p = 0.0001) (Table 2).

## Discussion

Vaccination is the method of choice to control AIV in poultry industry in many countries. Fast, efficient, and inexpensive DIVA test has a vital role in the success of this strategy. To address this issue, we expressed the M2e peptide as a recombinant M2e-MBP protein in *E.coli*. After purification by affinity chromatography, its specificity to detect M2e antibodies was confirmed by both Western blotting and ELISA. Synthetic M2e peptide has been used previously in chickens [22]. Application of M2e as recombinant protein, instead of as a peptide, has dual benefits. Recombinant M2e provides a continuous access to an inexpensive reagent for a large scale screening. The second benefit of recombinant M2e protein is the opportunity of demonstrating the M2e-based assay specificity by another method, in this case Western blotting, which is not possible when M2e is used as a peptide.

The expressed recombinant M2e-MBP protein had an expected size of 45.1 kd corresponded to 2.6 kd M2e and 42.5 kd MBP. Another minor protein of 42.5 kd corresponding to truncated M2e-MBP, was also co-purified with M2e-MBP and was not possible to remove from the preparation of M2e-MBP by additional purification.

Western blotting recognised the M2e-MBP protein of 45.1 kd length only by antisera to live AIV whereas antisera to inactivated H5N1 did not react with the M2e-MBP. This reaction in Western blotting was demonstrated to be due to M2e only and not due to MBP.

The comparison of M2e-MBP and peptide ELISA using reference sera indicted that M2e-MBP antigen was equally capable of detecting M2e positive sera without any significant background noise, or non-specific reaction. All sera that were positive by peptide ELISA were also positive in M2e-MBP ELISA, and in full agreement with the results obtained by M2e-MBP based Western blotting, indicated that M2e expressed as fusion M2eMBP protein is antigenically functional. The recombinant M2e-MBP was expressed in large quantities and allowed sera to be tested for M2e antibodies at a relatively high dilution of 1/100. The signal to noise ratio in ELISA was significantly different and allowed clear differentiation of M2e positive reference sera.

UP to now, M2e peptide has been expressed in *Salmonella*, *Pichia pastoris*, plants and viruses for the purpose of studying the protective immunity induced by the M2e [29], [30], [31]. Only in one study the M2e was expressed as GST fusion protein in *E. coli* and used to analyse the antibody response to the swine influenza virus M2e [32]. Application of efficient and high productive *E.coli* platform in this study provides the opportunity of production of a large amount of recombinant M2e protein in a short period of time. Further dilution of the producd protein (1/100 dilution, because of high ratio in this study) as a result of high recombinant protein yield in *E.coli* platform significantly decreases the non-specific resulting in more accurate DIVA test.

The comparison of the results of M2e-MBP ELISA and HI (HA-based) tests demonstrated the excellent ability of M2e-MBP antigen to discriminate between antibodies produced against live virus challenge and killed virus vaccination for DIVA serological test. M2e-MBP ELISA was highly sensitive to live virus infection as the ELISA OD of vaccinated chickens greatly increased by more than 8 time when vaccinated chickens were infected with the live AIV virus (Figure 5).

Furthermore, the robustness of the method reinforced by large scale M2e-MBP ELISA in differentiation of infected chickens from non-infected and vaccinated chickens (Figure 5). With negative field sera the background noises in M2e MBP ELISA were however higher. In order to reduce the of biases of non-specific binding, each serum sample was tested in duplicate in the presence and absence of M2e MBP antigen and background noise subtracted from OD obtained on M2e MBP. This non-specific binding of a percentage of field sera is largely independent of the antigen used for coating as shown by others [23] and also was occurred in M2e peptide ELISA.

The newly designed M2e-MBP ELISA system using as a DIVA tool has some limitations in its application in old chicken and the serum samples that were haemolysed or lipemic. Some of the field serum samples from old flocks (older than 44 weeks) had different degrees of non-specific reactions with M2e-MBP antigen. Western blot analysis using MBP purified fusion protein revealed that these samples had different reactivity just with MBP fusion protein but not with M2e.

Also some of the serum samples from layer or free range chicks had non-specific reactions to MBP fusion protein and/or just the ELISA plates alone. To overcome these problems using fresh, non-haemolysed and preferably non-lipemic serum samples would be an advantageous. Where it is necessary the results can be confirmed by Western blotting or subtracting the MBP value from the whole antigen. Second critical item in reliability of the M2e-MBP ELISA is purity of the antigen, in some cases during purification procedure of the antigen some of *E.coli* proteins could contaminate purified protein, and these residues will react with chicken antibodies, increasing the background of the tested samples in ELISA. Regarding some non-specific binding encountered in indirect M2e-MBP ELISA, being either due to the nature of the antigen or the properties of the chicken serum, we suggest that the use of a competitive or blocking ELISA for the detection of M2e antibodies might be an approach to reduce non-specific reactions with chicken sera.

H5N1-originated recombinant M2e-MBP protein or synthetic M2e peptide reacts with a wide range of other AIV strains antibodies such as H5N2, H9N2, H7N7, H11N6, etc. While the major surface influenza glycoproteins, HA and NA undergo major antigenic changes resulting in short-time effectiveness of available vaccines, the extracellular domain of matrix protein 2 (M2e) is strongly invariable and conserved. Therefore, vaccines based on extracellular domain of M2e are capable in inducing broad-spectrum immunity and inhibit virus replication up to 90–100% protection in mice and heterosubtypic immunity in pigs [16], [18]. In fact, the recent concept of a “universal influenza vaccine” relies on the conserved ectodomain of the influenza A protein [16], [18].

In addition to the above-mentioned advantages, extracellular domain of M2e opens a new vista in avian influenza management via DIVA test. Hydrophilic structure of M2e as well as its invariability and broad-spectrum reactivity show that M2e is an appropriate candidate for both vaccine production and DIVA test. The high performance of M2e subunit for DIVA test can be explained by considerable lower number of M2 molecules per virion (20–60) comparing to HA and NA. Due to larger amount of HA molecule per virion. HA can induce a very strong immune response in both vaccinate (killed virus) and infected chickens. In contrast, the amount of M2e subunit in vaccine is very low, and immune response can be detected after live virus infection as presented in Figure 4.
