# Expression of Concern: Recombinant M2e Protein-Based ELISA: A Novel and Inexpensive Approach for Differentiating Avian Influenza Infected Chickens from Vaccinated Ones

**DOI:** 10.1371/journal.pone.0250485

**Published:** 2021-04-15

**Authors:** The PLOS ONE Editors

After this article [1] was published, concerns were raised about results reported in Figure 1. When levels are adjusted to visualize the background in this figure:

- Figure 1B, lanes 2, 6, and 7 appear similar.
- Figure 1C, lanes 1, 2, 3, and 4 appear similar.
- Figure 1C, lanes 5 and 7 appear similar.

The authors noted that duplications may have been due to errors made when negative data were being prepared for the manuscript. They provided the available raw image data for Figure 1 from the original experiments; these images were not of sufficient quality to provide clear information as to the issues or results. However, lab records and image data for replication experiments (S1–S3 Files) lend support for the overall results that none of the Figure 1B or 1C samples yielded positive MBP blotting results, and that the Scotland, Vietnam, Turkey, and Denmark sera, but no other samples tested, reacted with M2e-MBP.

In following up on this issue, the authors provided the following clarifications about the western blot protocol:

- Purified proteins were run in an IPG big well in discontinuous (12.5%) SDS-PAGE gels. Before the blot transfer, a small vertical strip of the gel representing a single lane was cut and stained with Coomassie Blue to check for protein separation on the gel. After the transfer, the authors checked for protein transfer by staining gels with Coomassie Blue and checking blots for transfer of pre-stained protein markers. The blot membranes were not stained or reprobed to obtain control data demonstrating the amount of transferred protein on each blot strip.
- After the blocking step, the membrane was cut into strips and each lane was placed into a well of a 12-well tray. Blot signal was developed separately for each strip, and the authors tried to incubate each strip for approximately the same amount of time (~10 minutes). Membranes were then dried and imaged using a digital camera. Figure panels were assembled using multiple membrane strips that were developed separately but within the same experiment.

The results should be interpreted with caution in light of these aspects of the study design, particularly in regard to negative results and comparisons across blots.

The *PLOS ONE* Editors issue this Expression of Concern to notify readers of the above issues and to provide the available replication data for the experiments of concern.

## Supporting information

S1 File Compilation of lab records showing replication data. (PDF)

S2 File Compressed File 1–1. High-resolution image files of raw data.
(ZIP)

S3 File Compressed File 1–2. High-resolution image files of raw data.
(ZIP)
