# Supplementary material for: Expression of Concern: Recombinant M2e Protein-Based ELISA: A Novel and Inexpensive Approach for Differentiating Avian Influenza Infected Chickens from Vaccinated Ones
Source: PLoS One. 2021 Apr 15;16(4):e0250485. doi: 10.1371/journal.pone.0250485 (PMC8049298; doi:10.1371/journal.pone.0250485)
Supplement: S1 File — (PDF) [file pone.0250485.s001.pdf]

Description of the photos and the scanned pages.
In following photos, we describe the links between the original photos and the strips at the figure 1 of the published paper. The descriptions for each original photos are highlighted in yellow and blue at the pages of 11, 12, 13 and 14. Higher resolution of photos are provided at different files.

Page 39:

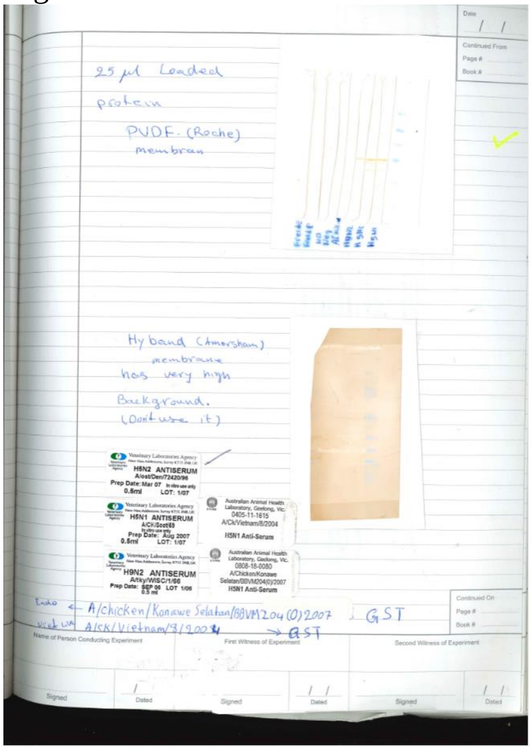

The standard antibodies and the first Western blotting assay using the positive and negative samples.

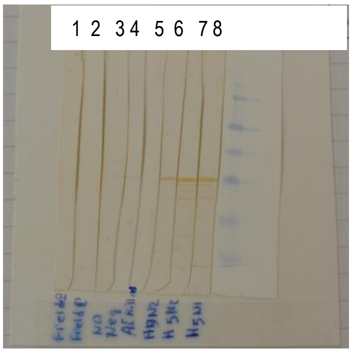

1, 2, 3 and 4 negative field samples, 5 killed vaccine
6 , 7 and 8 positive serum sample.
H5N1 is antiserum to live A/Chicken/Scotland/1959
H9N2 is antiserum to live A/Turkey/Wisconsin/1/1966
H5N2 is antiserum to live A/Ostrich/Denmark/72420/1996

Page 78, experiment at 12.12.08

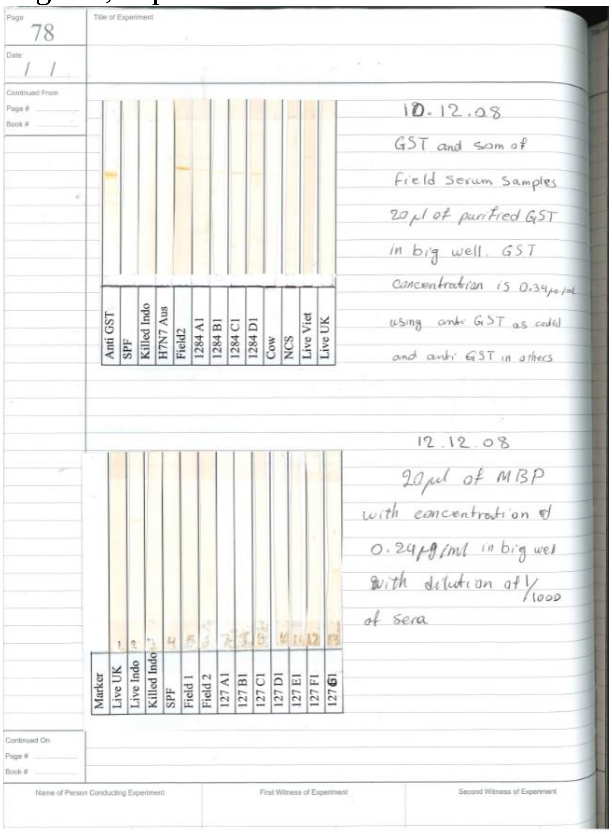

Field serum samples in MBP Western blotting, all negative

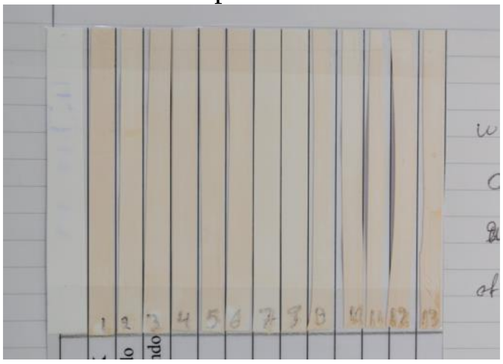

1) Live UK H5N1 (A/Chicken/Scotland/1959)
2) Live Indonesia H5N1 (9)
3) Killed Indonesia H5N1 (2)
4) SPF

5-13) all field serum samples from commercial chicken farms (no vaccine no infection)

Page 84

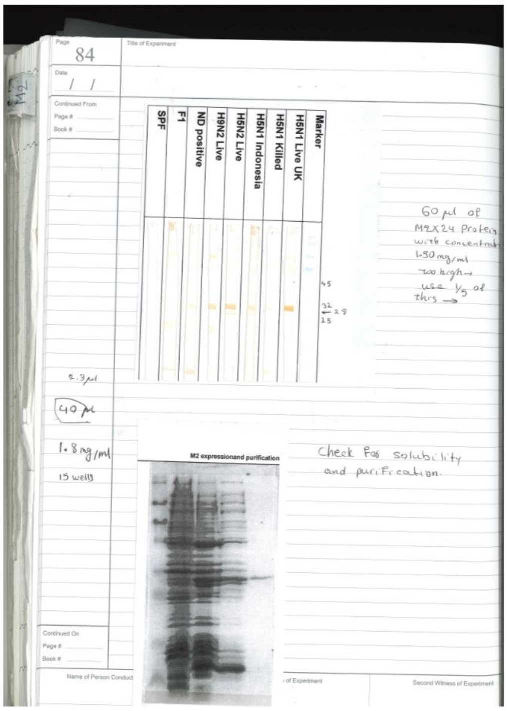

Different serum samples in M2e-MBP

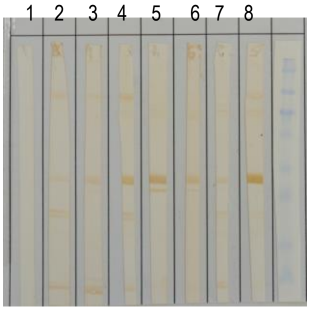

1) SPF 2
2) Field 1
3) NDV positive
4) Live H9N2 A/Turkey/Wisconsin/1/1966
5) Live H5N2 A/Ostrich/Denmark/72420/1996
6) Live Indonesia H5N1 (10)
7) Killed Indonesia H5N1 (107)
8) Live H5N1 A/Chicken/Scotland/1959

Page 93

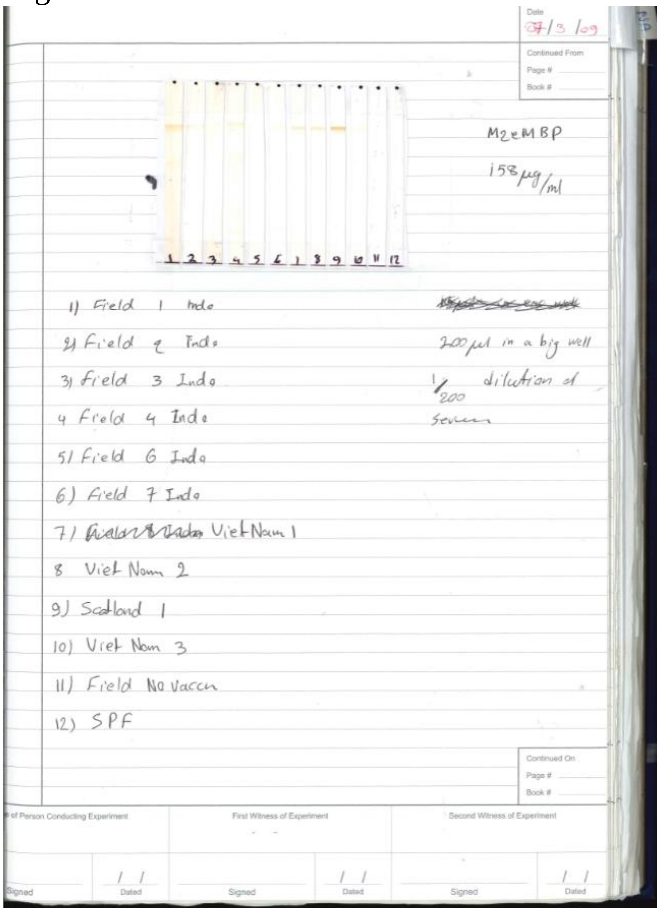

M2e MBP in field serum samples from vaccinated chicken from Indonesia. (Vaccinated no infection, PCR negative farms)

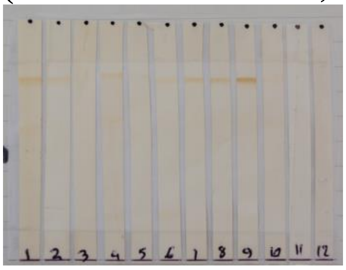

1-6) Serum samples from H5N1 vaccinated and PCR positive chicken from Indonesia
7) Live A/Ck/Viet Nam/8/2004 (H5N1) sample 1
8) Live A/Ck/Viet Nam/8/2004 (H5N1) sample 2
9) Live H5N1 A/Chicken/Scotland/1959
10) Live A/Ck/Viet Nam/8/2004 (H5N1) sample 3
11) Field Australia no vaccine no infection
12) SPF

Page 94:

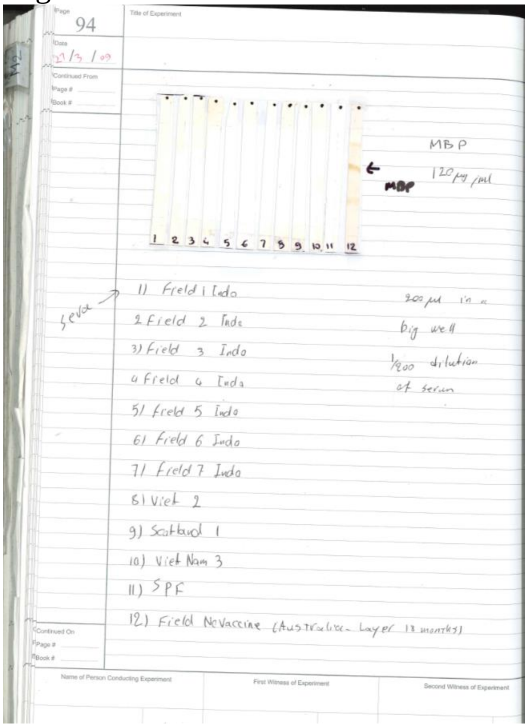

Same experiment as Page 93 on MBP carrier protein

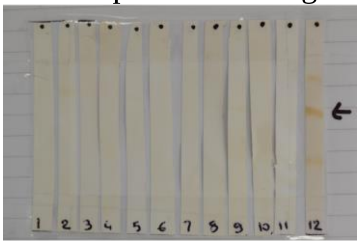

1-7) Serum samples from H5N1 vaccinated and PCR positive chicken from Indonesia
8) Live A/Ck/Viet Nam/8/2004 (H5N1) sample 2
9) Live H5N1 A/Chicken/Scotland/1959
10) Live A/Ck/Viet Nam/8/2004 (H5N1) sample 3
11) SPF
12) Field Australia no vaccine no infection

Page 95:

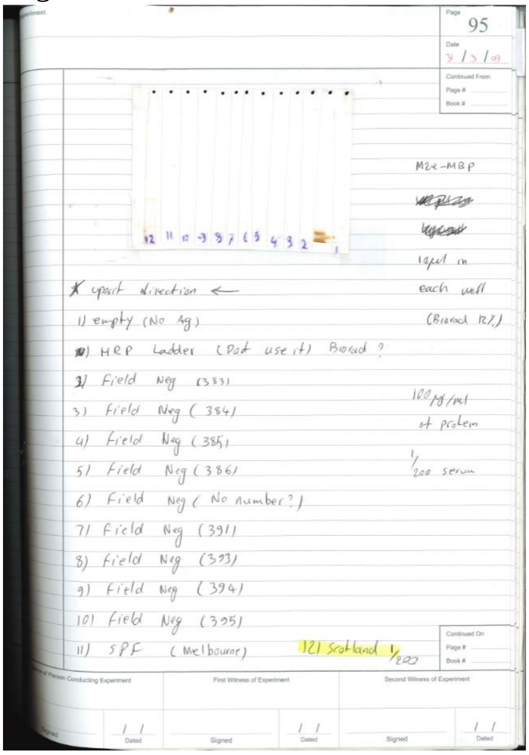

M2e MBP on negative field serum samples.

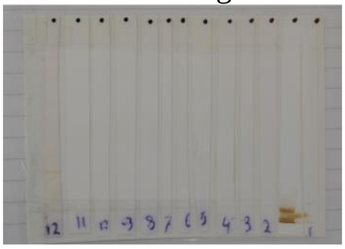

1-10) sera from No vaccine, no infection from commercial chicken farms.
11) SPF 2
12) Live H5N1 A/Chicken/Scotland/1959

Page 96:

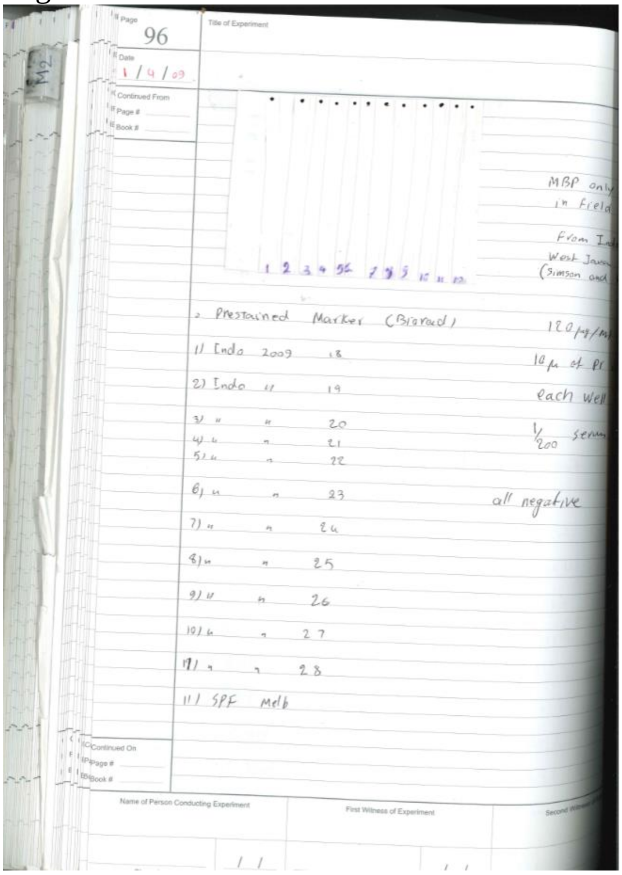

M2e MBP in second batch of field serum samples from vaccinated chicken from Indonesia. (Vaccinated no infection, PCR negative farms)

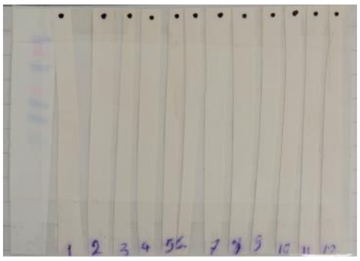

1-11) Vaccinated no infection, PCR negative farms
12) SPF 1

Page 97:

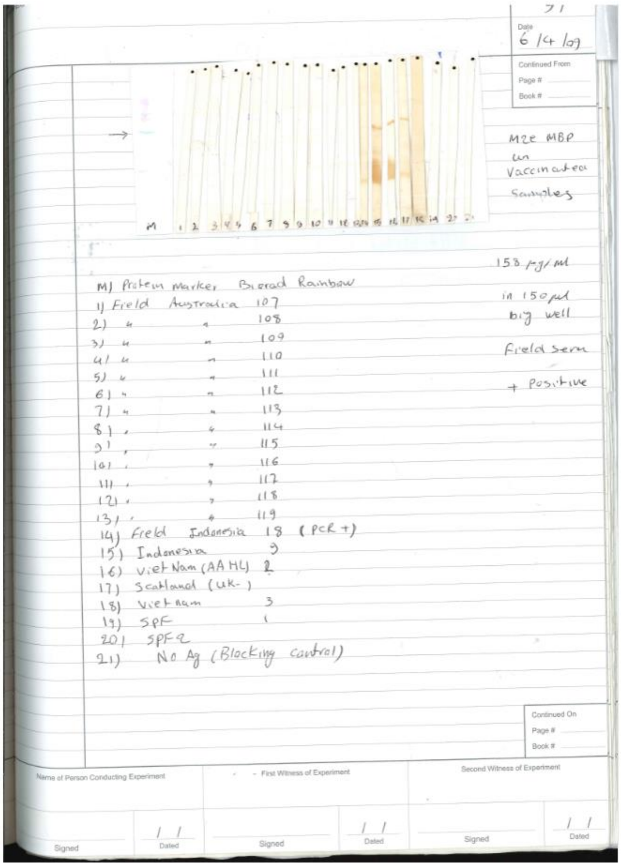
97

Date 6/4/09

Continued From Page # Book #

M2e MBP on Vaccinated Samples

153 µg/ml in 150µl big well

Field Serum + Positive

M) Protein Marker, Biorad Rainbow
1) Field Australia 107
2) " " 108
3) " " 109
4) " " 110
5) " " 111
6) " " 112
7) " " 113
8) " " 114
9) " " 115
10) " " 116
11) " " 117
12) " " 118
13) " " 119
14) Field Indonesia 18 (PCR +)
15) Indonesia 9
16) Viet Nam (AAHL) 2
17) Scotland (UK-)
18) Viet Nam 3
19) SPF 1
20) SPF 2
21) No Ag (Blocking control)

Continued On Page # Book #

Name of Person Conducting Experiment | First Witness of Experiment | Second Witness of Experiment

Signed / / Dated | Signed / / Dated | Signed / / Dated

M2e MBP in both positive and negative serum samples

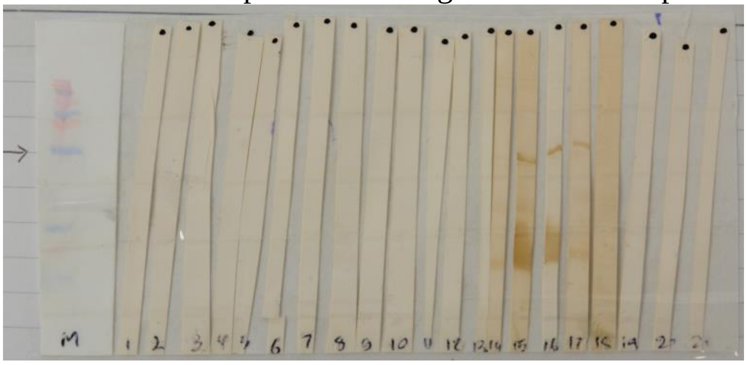

1-13) Serum samples from No vaccine, no infection from commercial chicken farms in Australia
14) Field serum sample from Indonesia (PCR positive farm)
15) Experimental serum sample H5N1 positive (Indonesia)
16) Live A/Ck/Viet Nam/8/2004 (H5N1) sample 2
17) Live H5N1 A/Chicken/Scotland/1959
18) Live A/Ck/Viet Nam/8/2004 (H5N1) sample 3
19) SPF 1
20) SPF 2
21) No antigen, blocking control

Page 98:

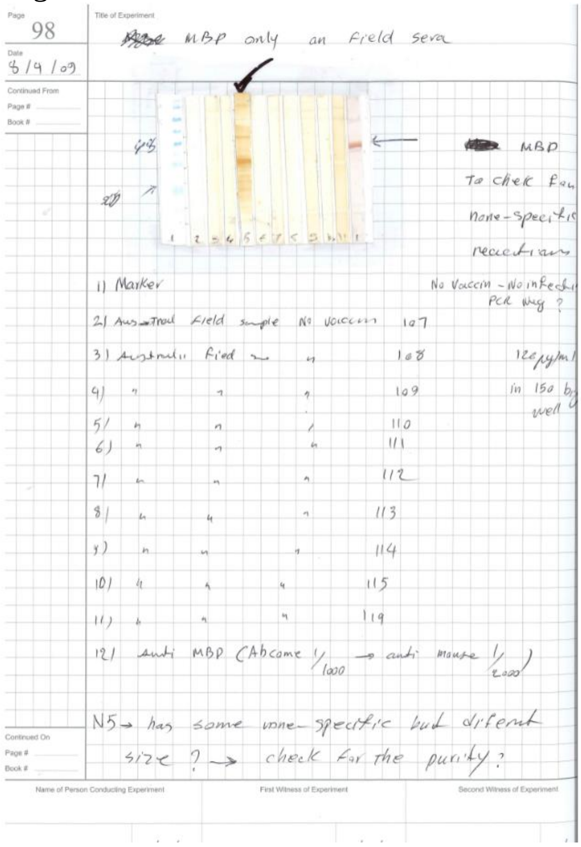

Page 98
Date 8/9/09
Title of Experiment: MBP only on Field sera

MBP
To check for none-specific reactions

No Vaccin - No infection
PCR neg ?

1) Marker
2) Australia Field sample No vaccin 107
3) Australia Field " " 108
4) " " " 109
5) " " " 110
6) " " " 111
7) " " " 112
8) " " " 113
9) " " " 114
10) " " " 115
11) " " " 119
12) anti MBP (Abcome 1/1000 → anti mouse 1/2000)

12 µg/ml in 150 by well

N5 → has some none-specific but different size ? → check for the purity ?

Continued From Page # Book #
Continued On Page # Book #
Name of Person Conducting Experiment | First Witness of Experiment | Second Witness of Experiment

MBP on field serum samples

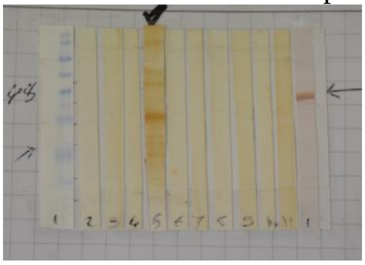

1-11) different batch of Serum samples from No vaccine, no infection from commercial chicken farms in Australia.
12) Developed with anti MBP antibody (Abcom) using anti mouse HRP

Page 100:

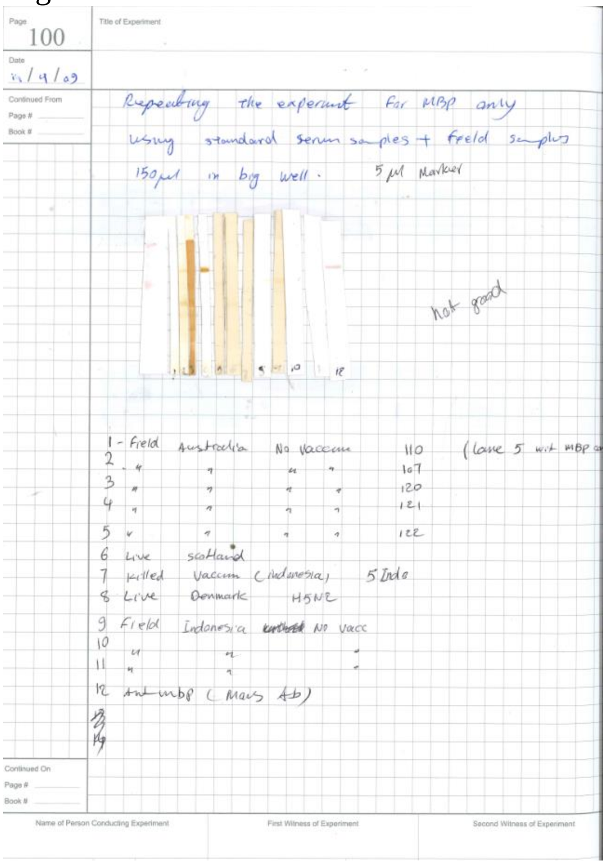
Page 100
Date 11/9/09
Title of Experiment
Continued From Page # Book #

Repeating the experiment for MBP only
using standard serum samples + field samples
150 µl in big well. 5 µl Marker

not good

1 - Field Australia No vaccine 110 (lane 5 with MBP [illegible]
2 " " " " 107
3 " " " " 120
4 " " " " 121
5 " " " " 122
6 Live Scotland
7 Killed Vaccine (Indonesia) 5 Indo
8 Live Denmark H5N2
9 Field Indonesia No vacc
10
11 " " "
12 Anti-mbp (Mous Ab)
13
14

Continued On Page # Book #
Name of Person Conducting Experiment | First Witness of Experiment | Second Witness of Experiment

Field serum samples and positive serum samples in MBP Western Blotting

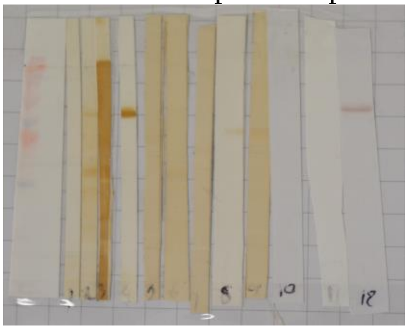

1-5) different batch of Serum samples from No vaccine, no infection from commercial chicken farms in Australia.
6)Live H5N1 A/Chicken/Scotland/1959
7) Killed Indonesia H5N1 (105)
8) Live H5N2 A/Ostrich/Denmark/72420/1996
9-11) Serum samples from H5N1 vaccinated and PCR negative chickens from Indonesia
12) Developed with anti MBP antibody (Abcom) using anti mouse HRP

Page 101:

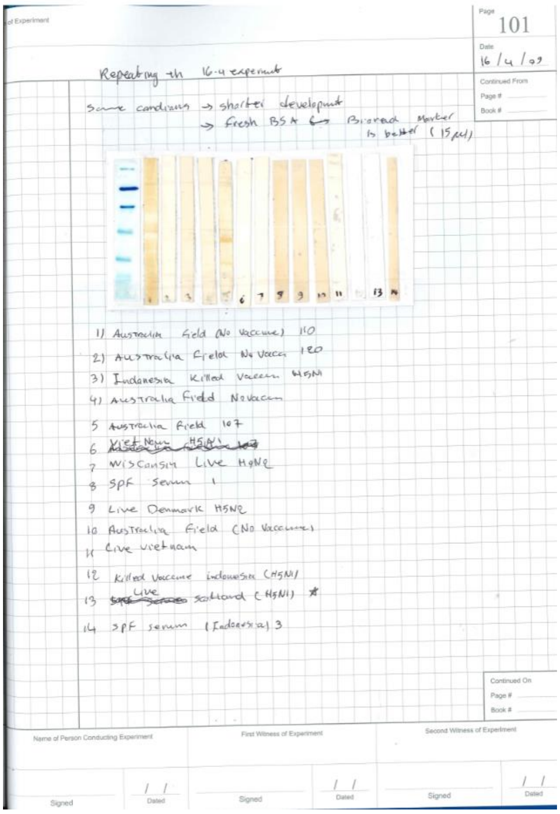

Last experiment on Western blotting on MBP only using positive and negative sera.

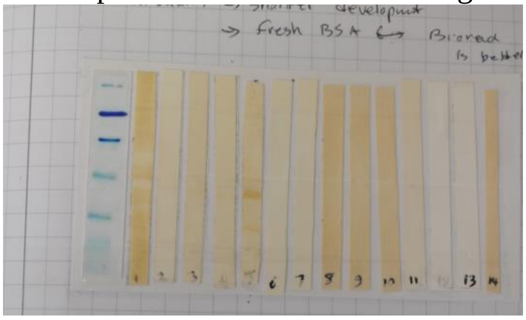

1) Serum sample from unvaccinated and un infected chicken from Australia
2) Serum sample from unvaccinated and un infected chicken from Australia
3) Serum samples from H5N1 vaccinated and PCR negative chickens from Indonesia
4) Serum sample from unvaccinated and un infected chicken from Australia
5) Serum sample from unvaccinated and un infected chicken from Australia, this strip is the strip number 6 at the figure 1C.
6) Live A/Ck/Viet Nam/8/2004 (H5N1) sample 2
7) Live H9N2 A/Turkey/Wisconsin/1/1966, this strip is the strip number 4 in figure 1C
8) SPF 1, this strip is the strip number 7 in figure 1C
9) Live H5N2 A/Ostrich/Denmark/72420/1996, this strip is the strip number 5 in figure 1C
10) Serum sample from unvaccinated and un infected chicken from Australia
11) Live A/Ck/Viet Nam/8/2004 (H5N1) sample 3, this strip is the strip number 3 in figure 1C

12) Killed vaccine Indonesia H5N1 commercial farm, this strip is the strip number 2 in figure 1C
13) Live H5N1 A/Chicken/Scotland/1959, this strip is the strip number 1 in figure 1C
14) SPF 3

Page 104:

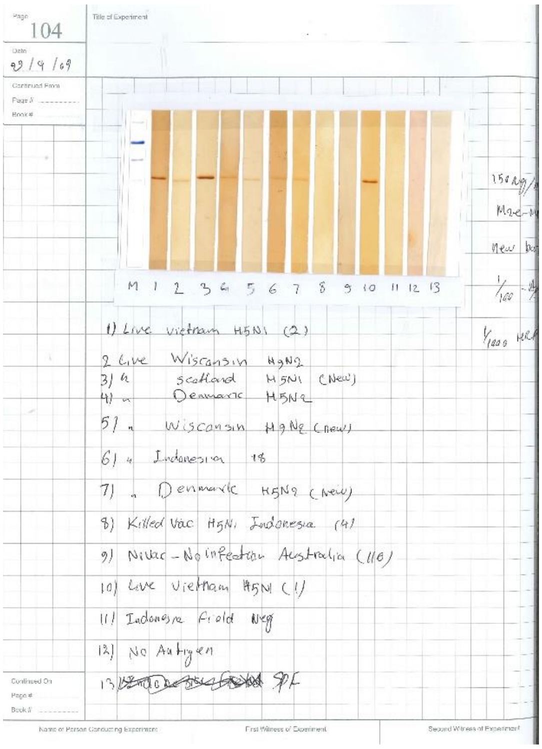
Page 104
Date 29/9/09

M 1 2 3 4 5 6 7 8 9 10 11 12 13

1) Live vietnam H5N1 (2)
2 Live Wisconsin H9N2
3) " Scotland H5N1 (New)
4) " Denmark H5N2
5) " Wisconsin H9N2 (new)
6) " Indonesia 18
7) " Denmark H5N2 (new)
8) Killed Vac H5N1 Indonesia (4)
9) Nivac - No infection Australia (116)
10) Live Vietnam H5N1 (1)
11) Indonesia Field Neg
12) No Antigen
13) SPF

M2e-MBP on positive and negative samples (same samples as page 101)

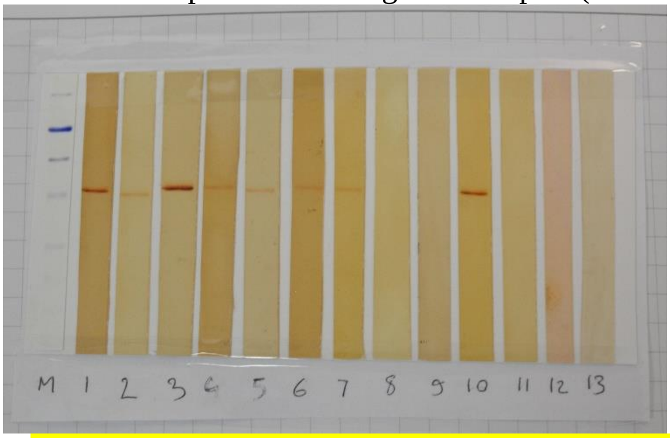

1) Live A/Ck/Viet Nam/8/2004 (H5N1) sample 2,

2) Live H9N2 A/Turkey/Wisconsin/1/1966, sample 2.

3) Live H5N1 A/Chicken/Scotland/1959, this strip is corresponding to the strip number 1 in figure 1B
4) Live H5N2 A/Ostrich/Denmark/72420/1996, sample 2
5) Live H9N2 A/Turkey/Wisconsin/1/1966, (Sample 1) this strip is the strip number 4 in figure 1B
6) Live Indonesia sample 18
7) Live H5N2 A/Ostrich/Denmark/72420/1996, (Sample 1) this strip is the strip number 5 in figure 1B
8) Killed vaccine Indonesia H5N1 commercial farm, this strip is the strip number 2 in figure 1B

9) Serum sample from unvaccinated and un infected chicken from Australia, this strip can be the strip number 6 in figure 1B
10) Live A/Ck/Viet Nam/8/2004 (H5N1) sample 2, this strip is corresponding to the strip number 3 in figure 1B
11) Serum sample from unvaccinated and un infected chicken from Australia, this strip can be the strip number 6 in figure 1B
12) SPF 3 this strip can be the strip number 6 in figure 1B

Page 103:

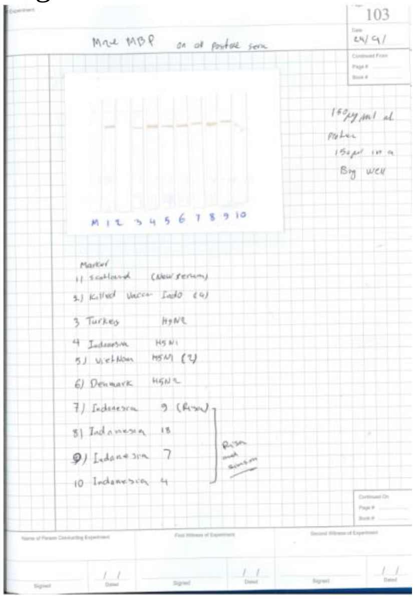

M2e MBP on all positive sera

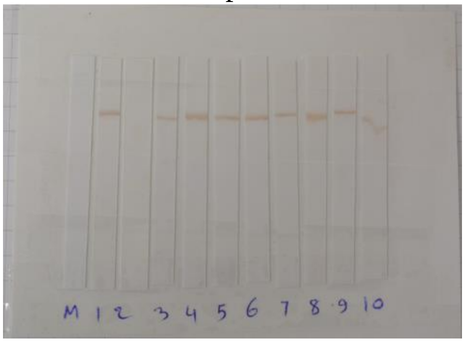

1) Live H5N1 A/Chicken/Scotland/1959
2) Killed vaccine Indonesia H5N1 commercial farm
3) Live H9N2 A/Turkey/Wisconsin/1/1966
4) Field serum sample from Indonesia (PCR positive farm)
5) Live A/Ck/Viet Nam/8/2004 (H5N1) sample 2
6) Live H5N2 A/Ostrich/Denmark/72420/1996
7) Experimental serum sample H5N1 positive (Indonesia) 9
8) Experimental serum sample H5N1 positive (Indonesia) 18
9) Experimental serum sample H5N1 positive (Indonesia) 7
10) Experimental serum sample H5N1 positive (Indonesia) 4
