# Supplementary figures and images for: Expression of Concern: Recombinant M2e Protein-Based ELISA: A Novel and Inexpensive Approach for Differentiating Avian Influenza Infected Chickens from Vaccinated Ones
Source: PLoS One. 2021 Apr 15;16(4):e0250485. doi: 10.1371/journal.pone.0250485 (PMC8049298; doi:10.1371/journal.pone.0250485)

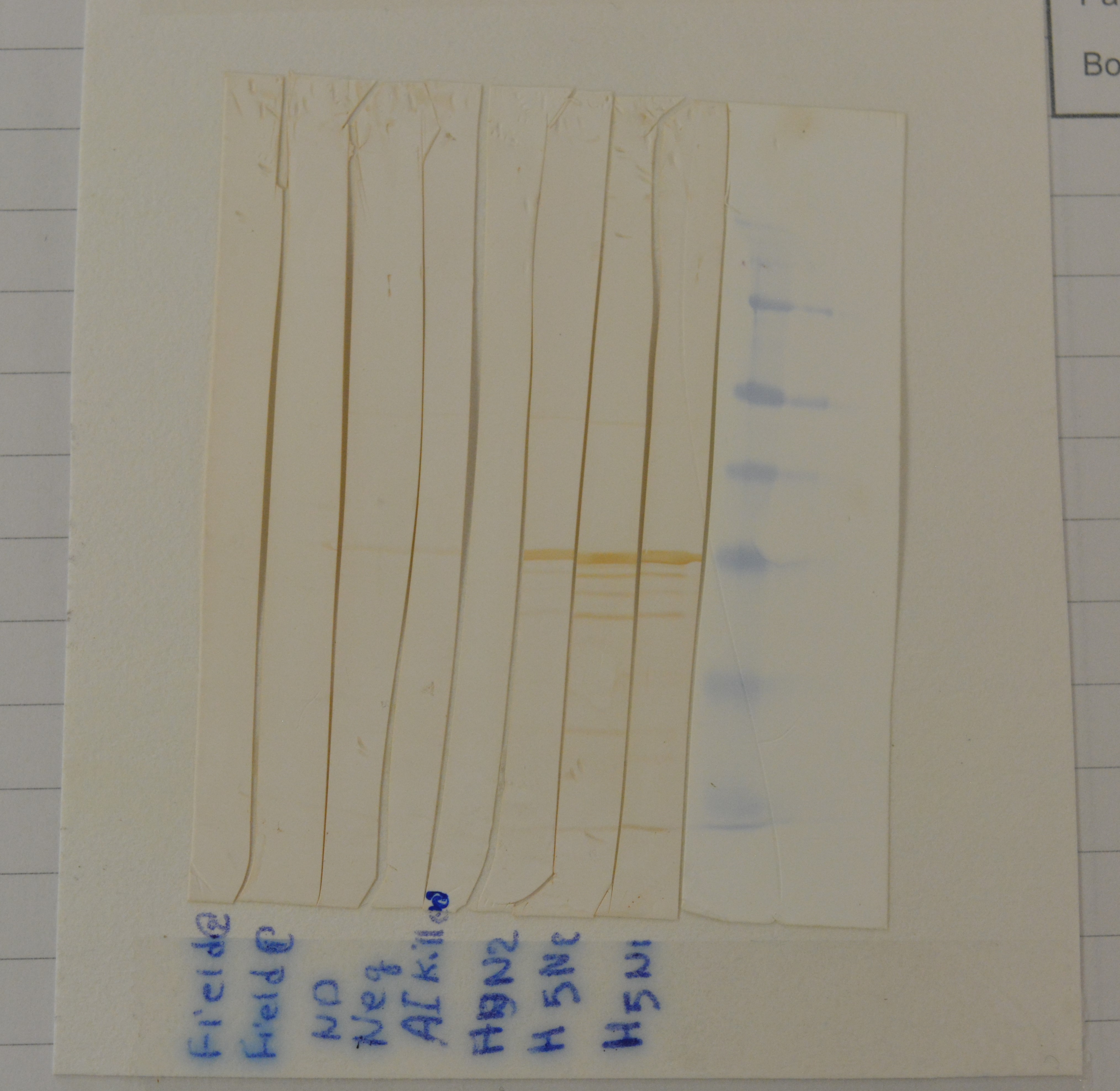

Supplement: S2 File — High-resolution image files of raw data. (ZIP) [file pone.0250485.s002.zip › P 39 Photo.jpg]

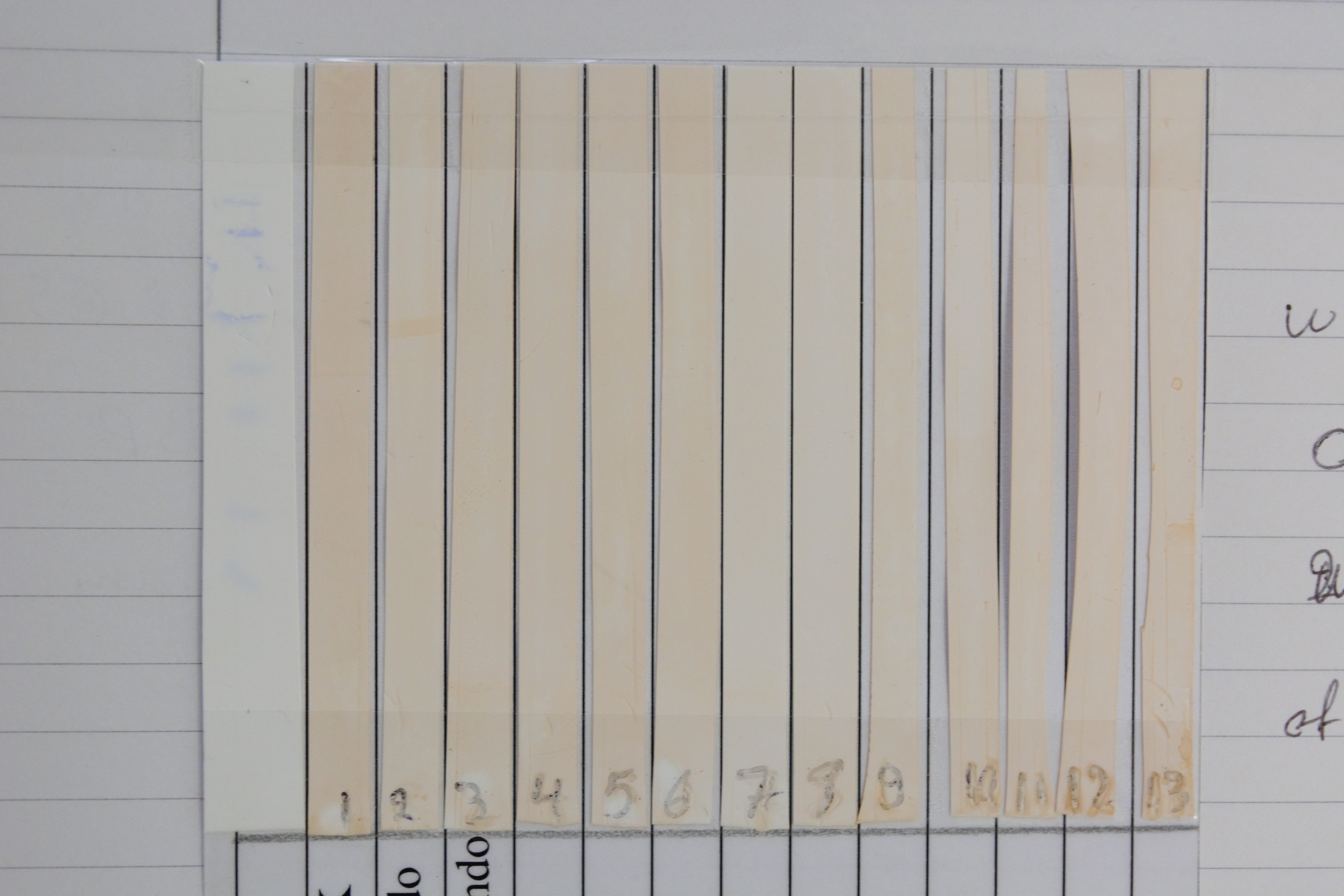

Supplement: S2 File — High-resolution image files of raw data. (ZIP) [file pone.0250485.s002.zip › P 78 Photo.jpg]

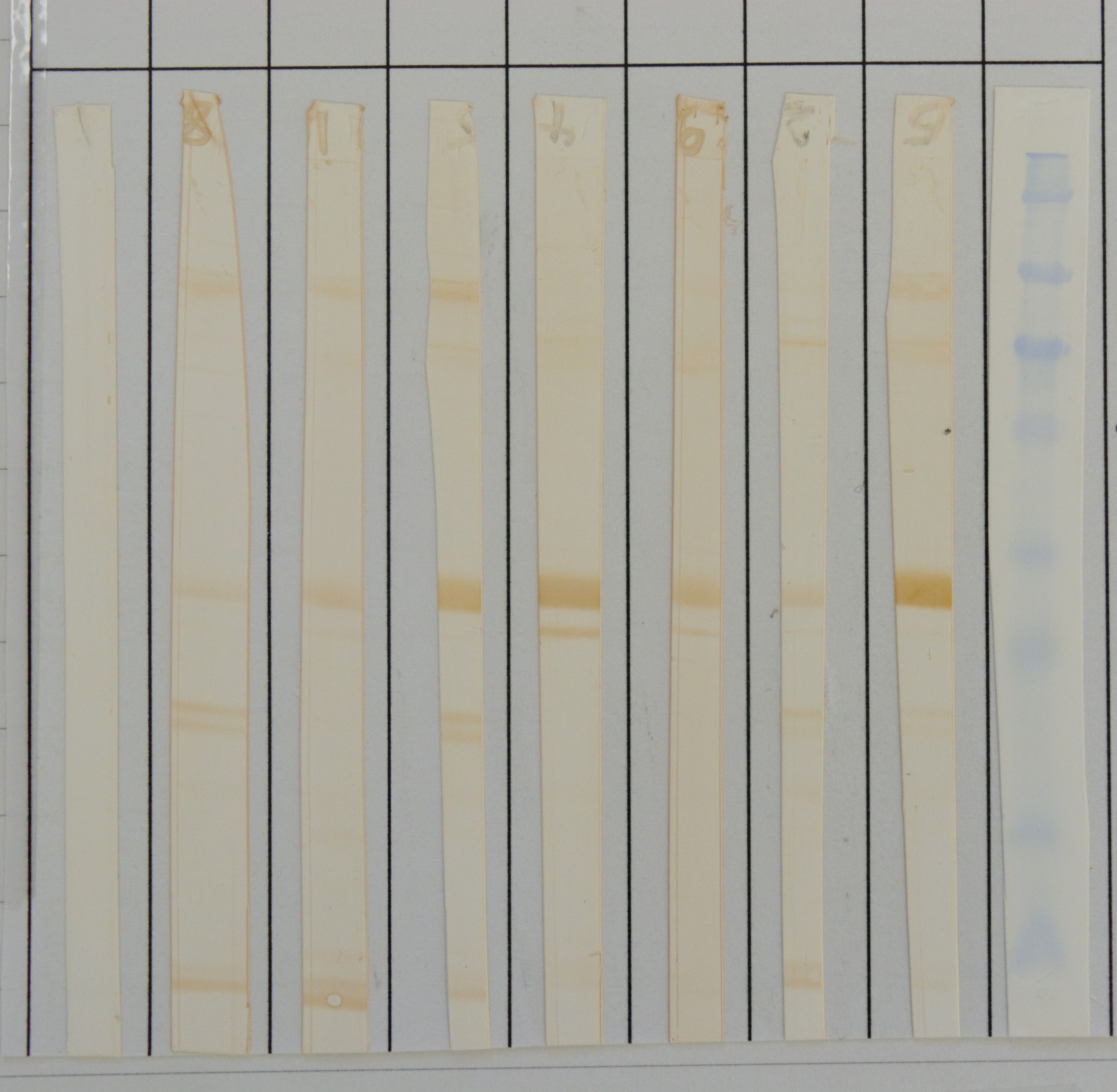

Supplement: S2 File — High-resolution image files of raw data. (ZIP) [file pone.0250485.s002.zip › P 84 Photo.jpg]

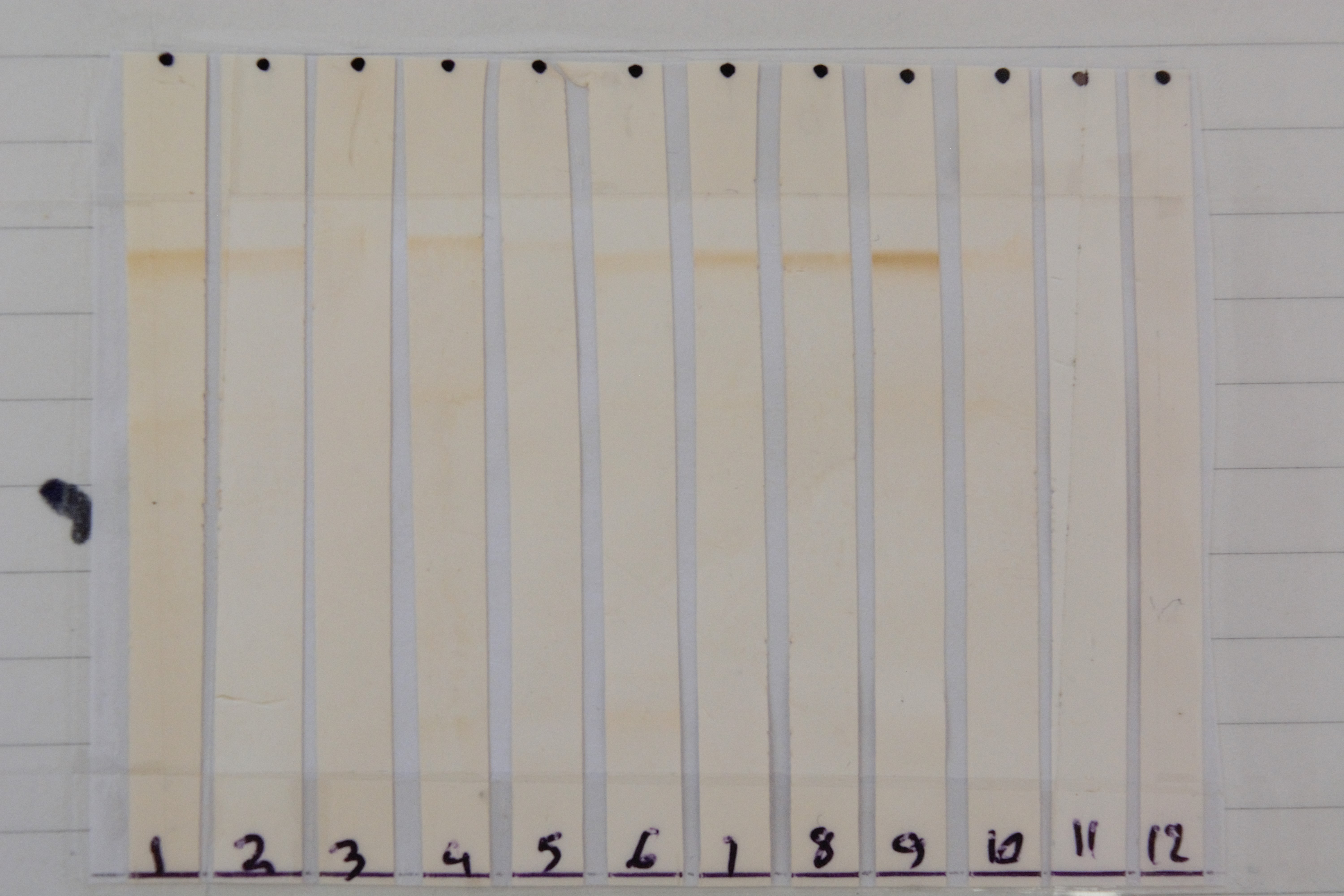

Supplement: S2 File — High-resolution image files of raw data. (ZIP) [file pone.0250485.s002.zip › P 93 Photo.jpg]

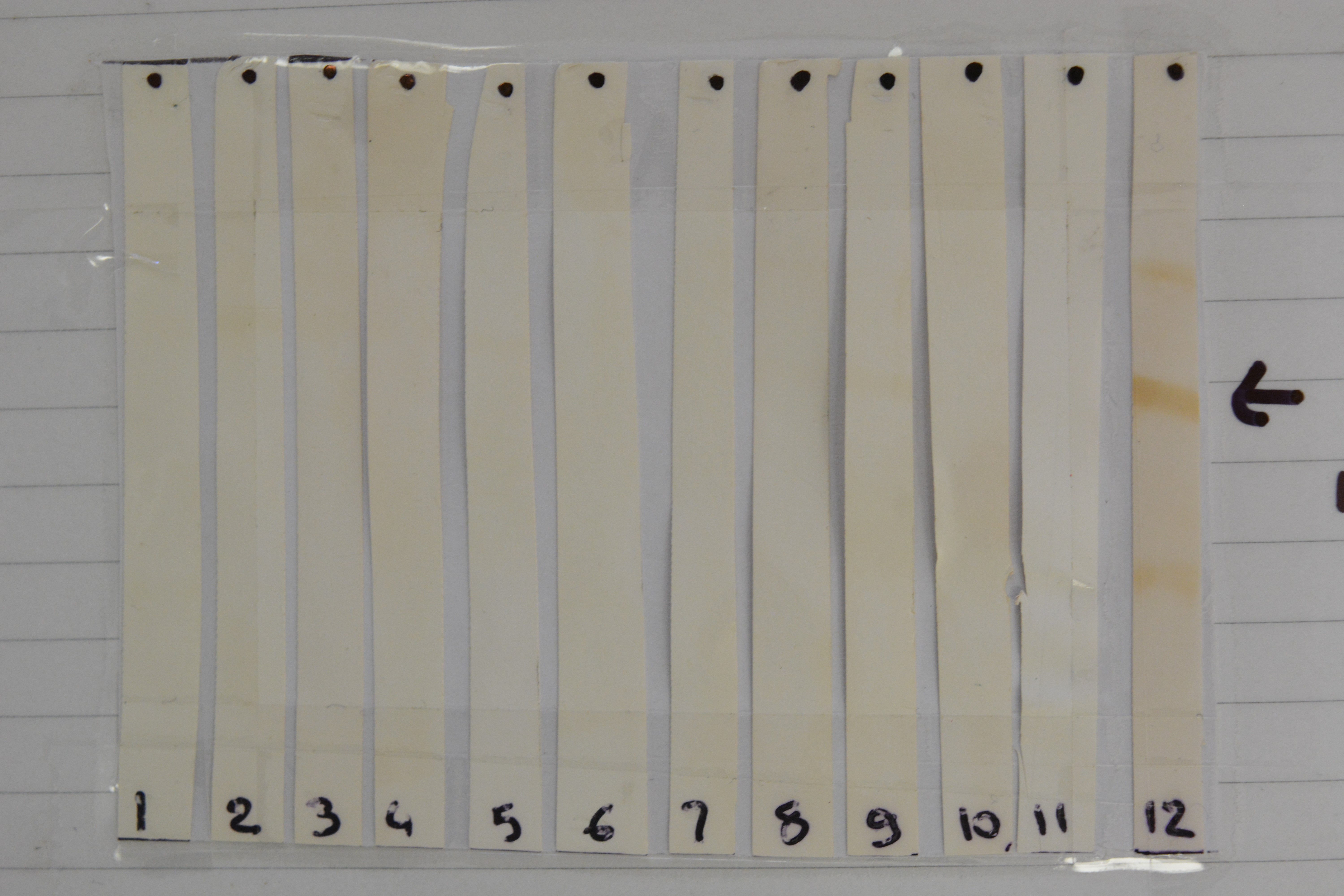

Supplement: S2 File — High-resolution image files of raw data. (ZIP) [file pone.0250485.s002.zip › P 94 Photo.jpg]

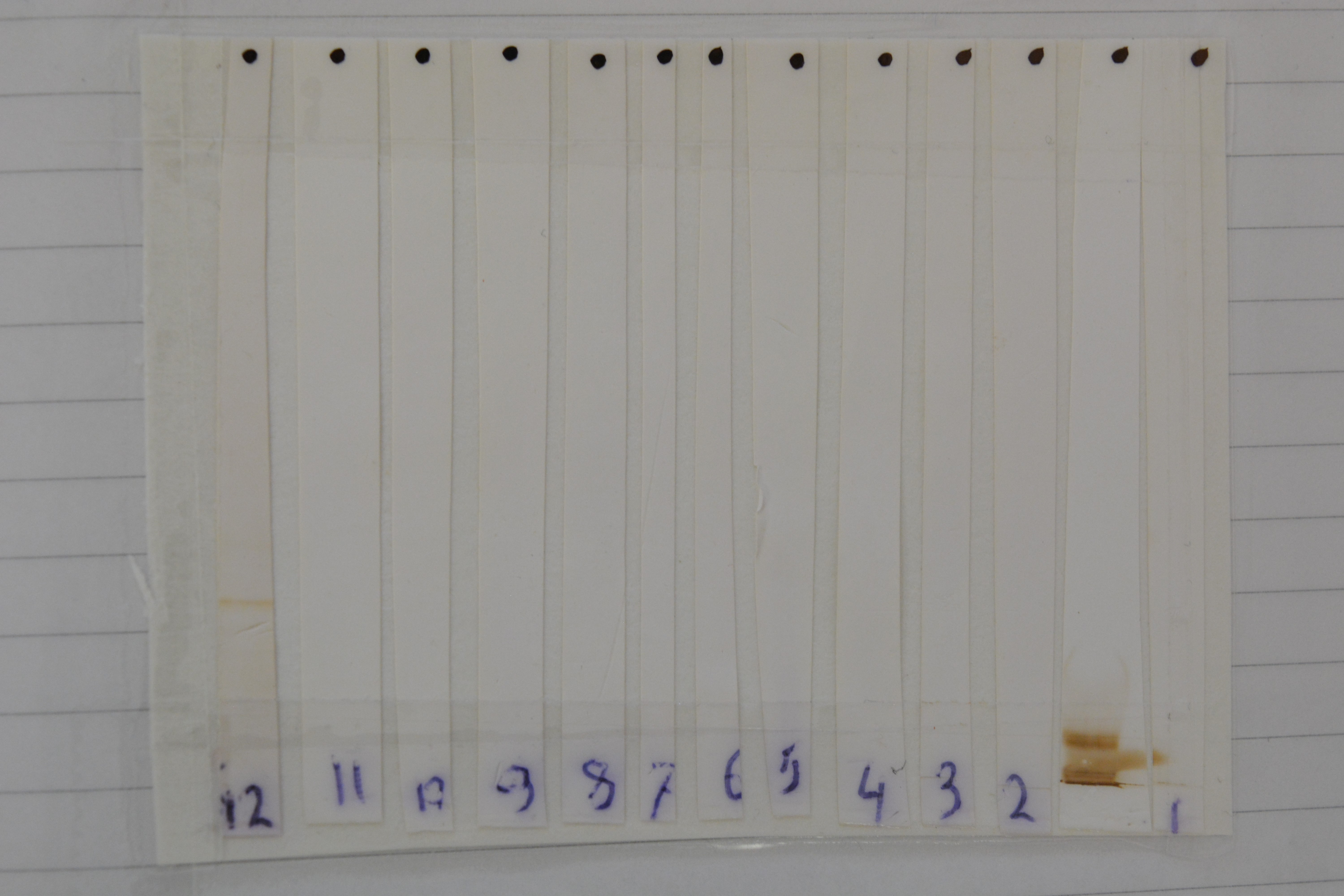

Supplement: S2 File — High-resolution image files of raw data. (ZIP) [file pone.0250485.s002.zip › P 95 Photo.jpg]

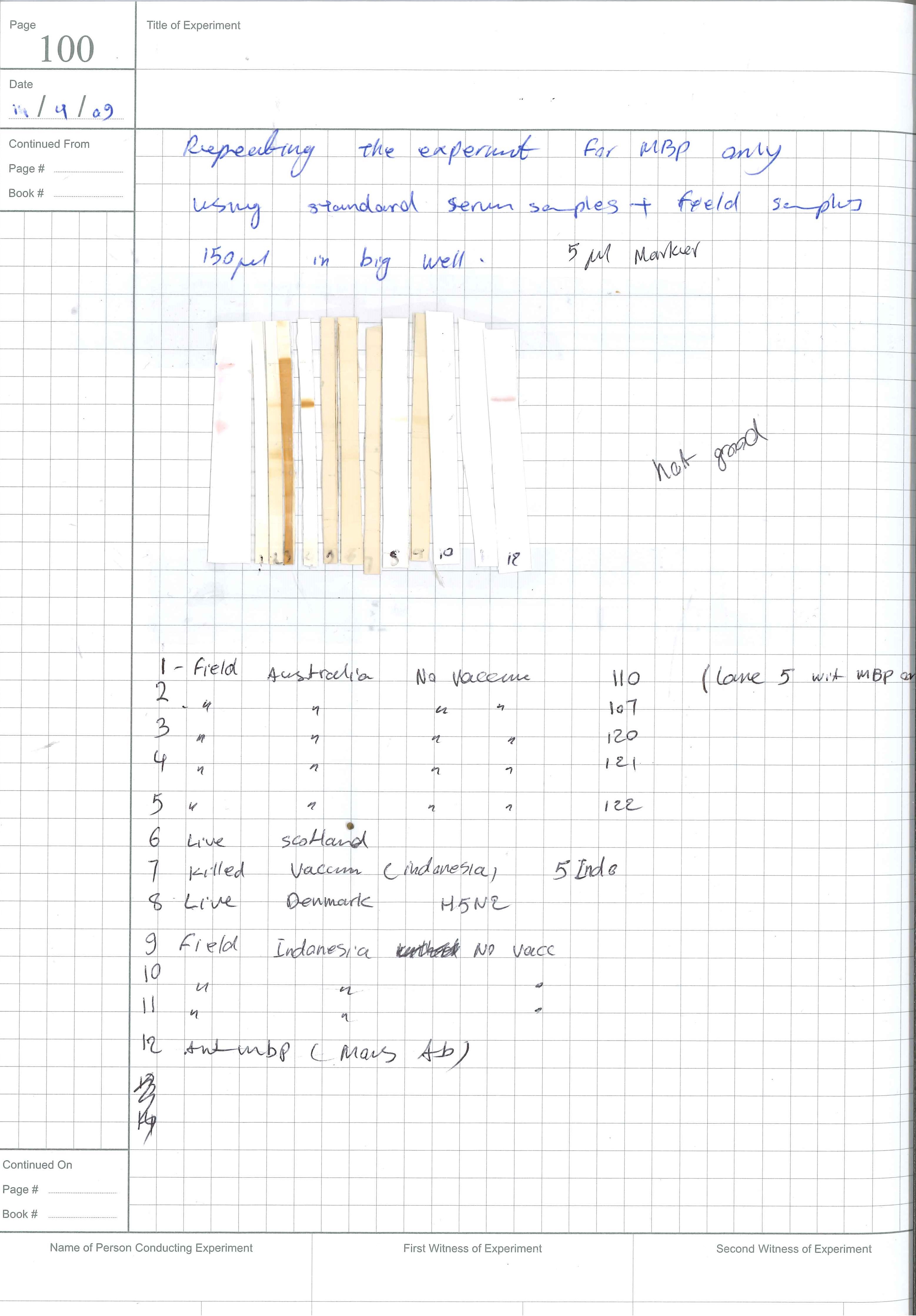

Supplement: S3 File — High-resolution image files of raw data. (ZIP) [file pone.0250485.s003.zip › P 100.jpg]

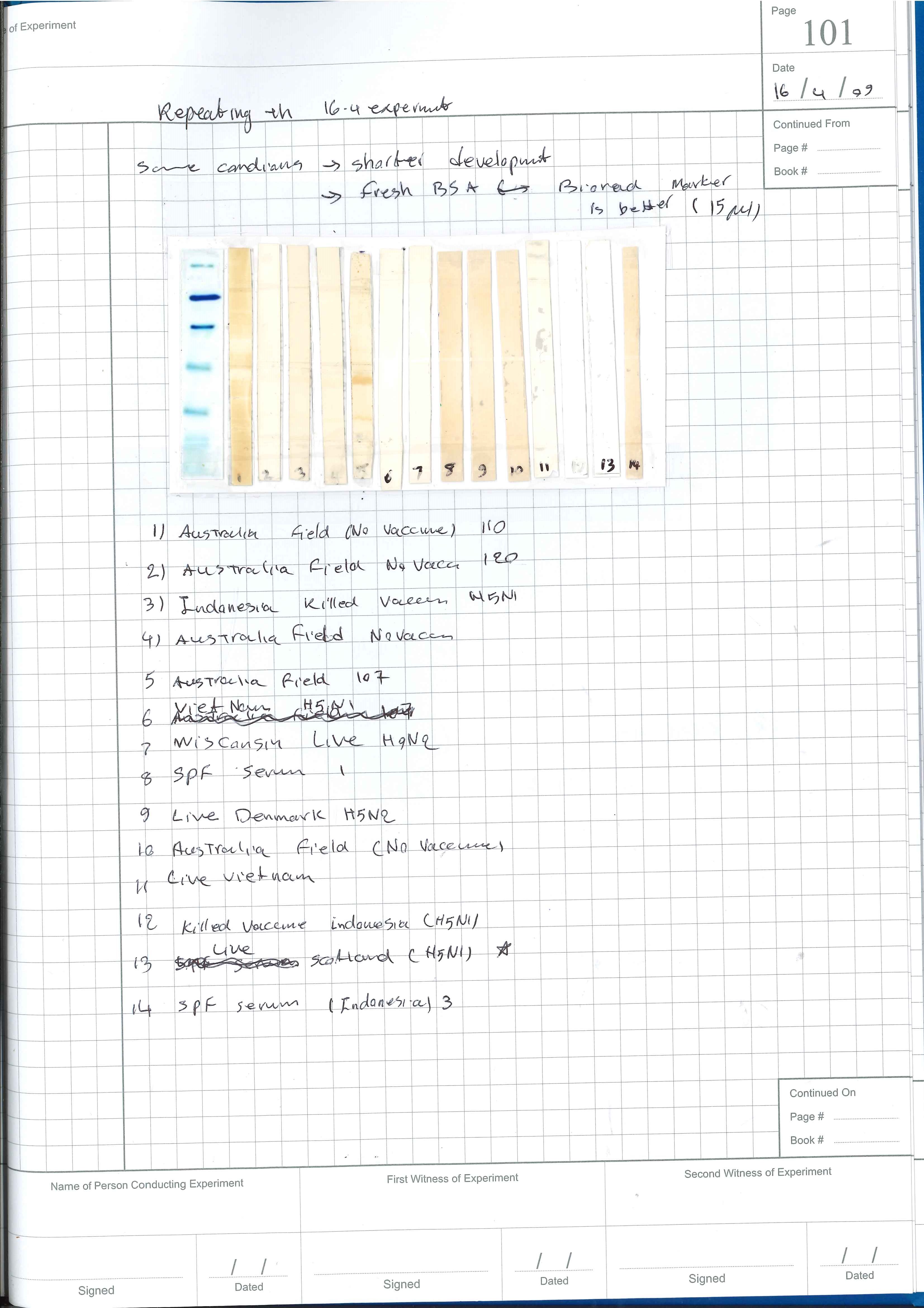

Supplement: S3 File — High-resolution image files of raw data. (ZIP) [file pone.0250485.s003.zip › P 101.jpg]

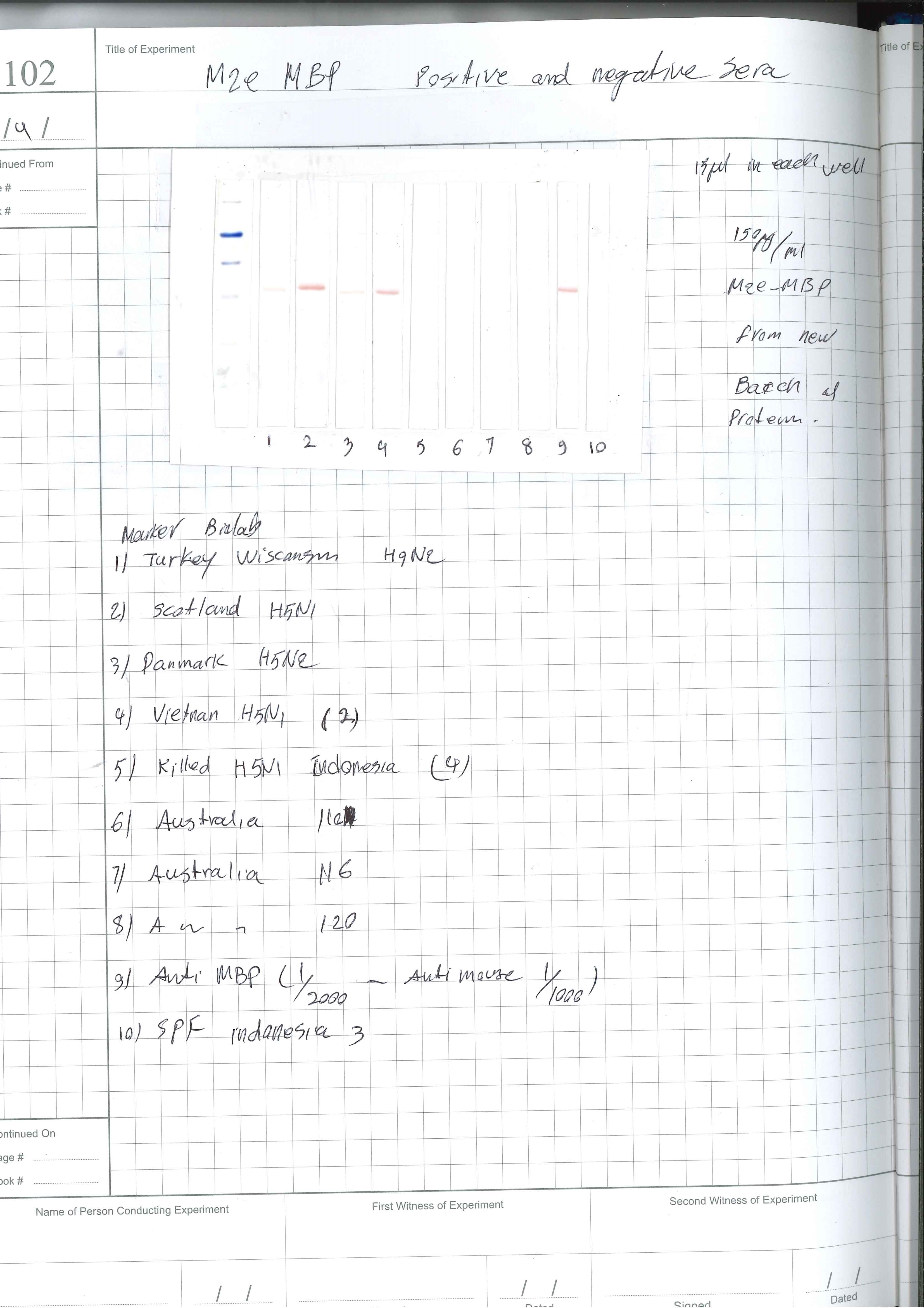

Supplement: S3 File — High-resolution image files of raw data. (ZIP) [file pone.0250485.s003.zip › P 102.jpg]

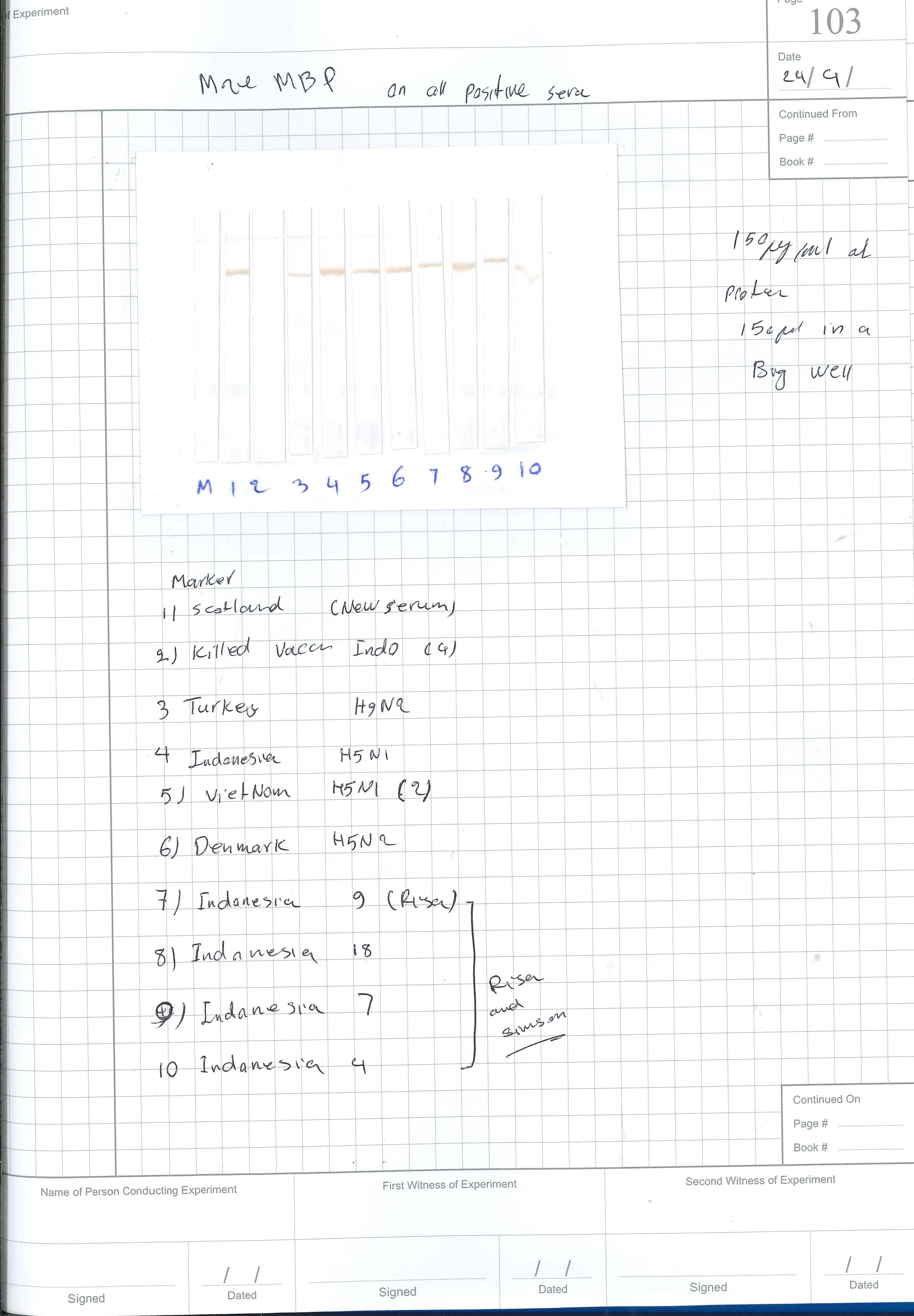

Supplement: S3 File — High-resolution image files of raw data. (ZIP) [file pone.0250485.s003.zip › P 103.jpg]

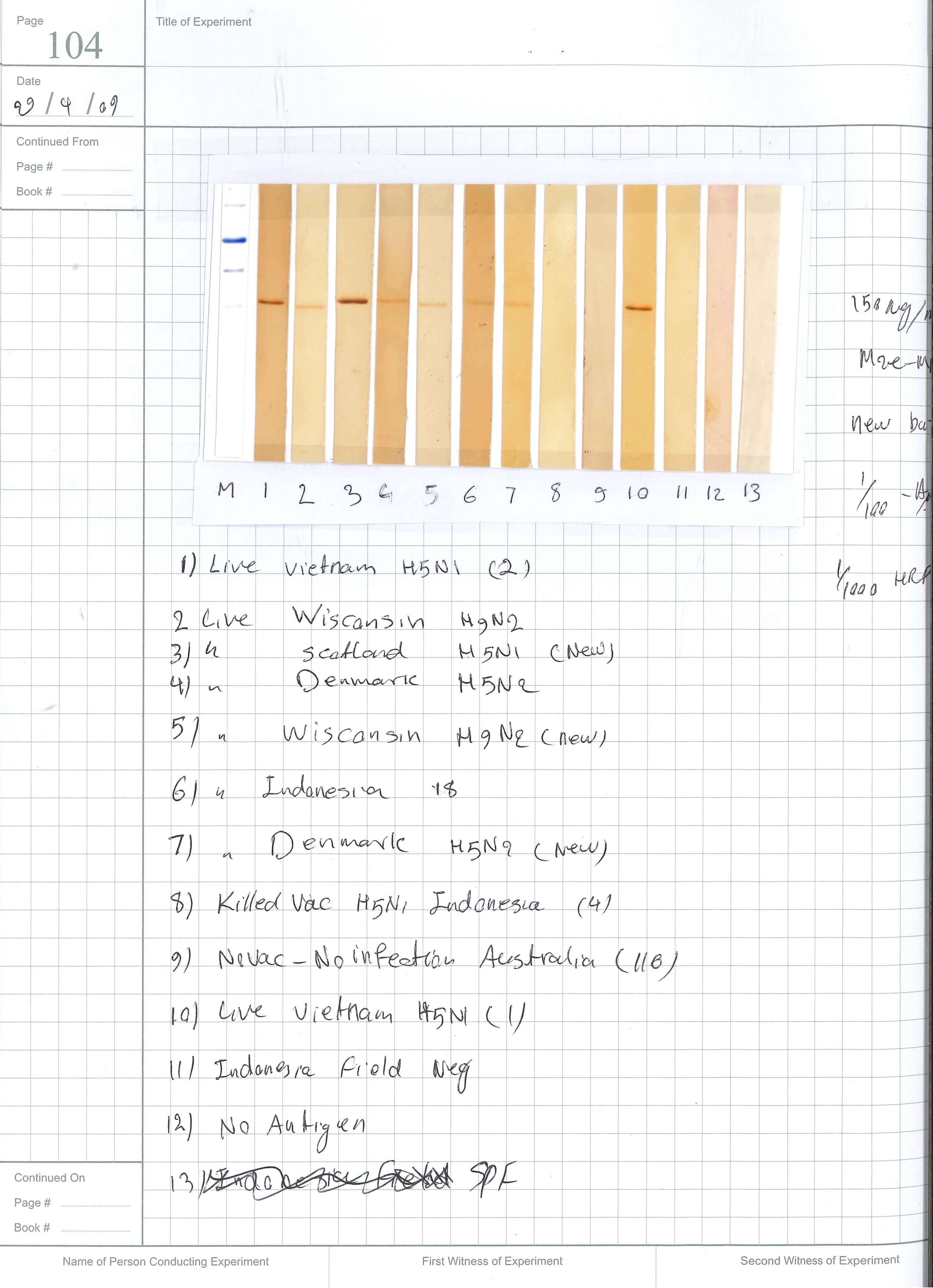

Supplement: S3 File — High-resolution image files of raw data. (ZIP) [file pone.0250485.s003.zip › P 104.jpg]

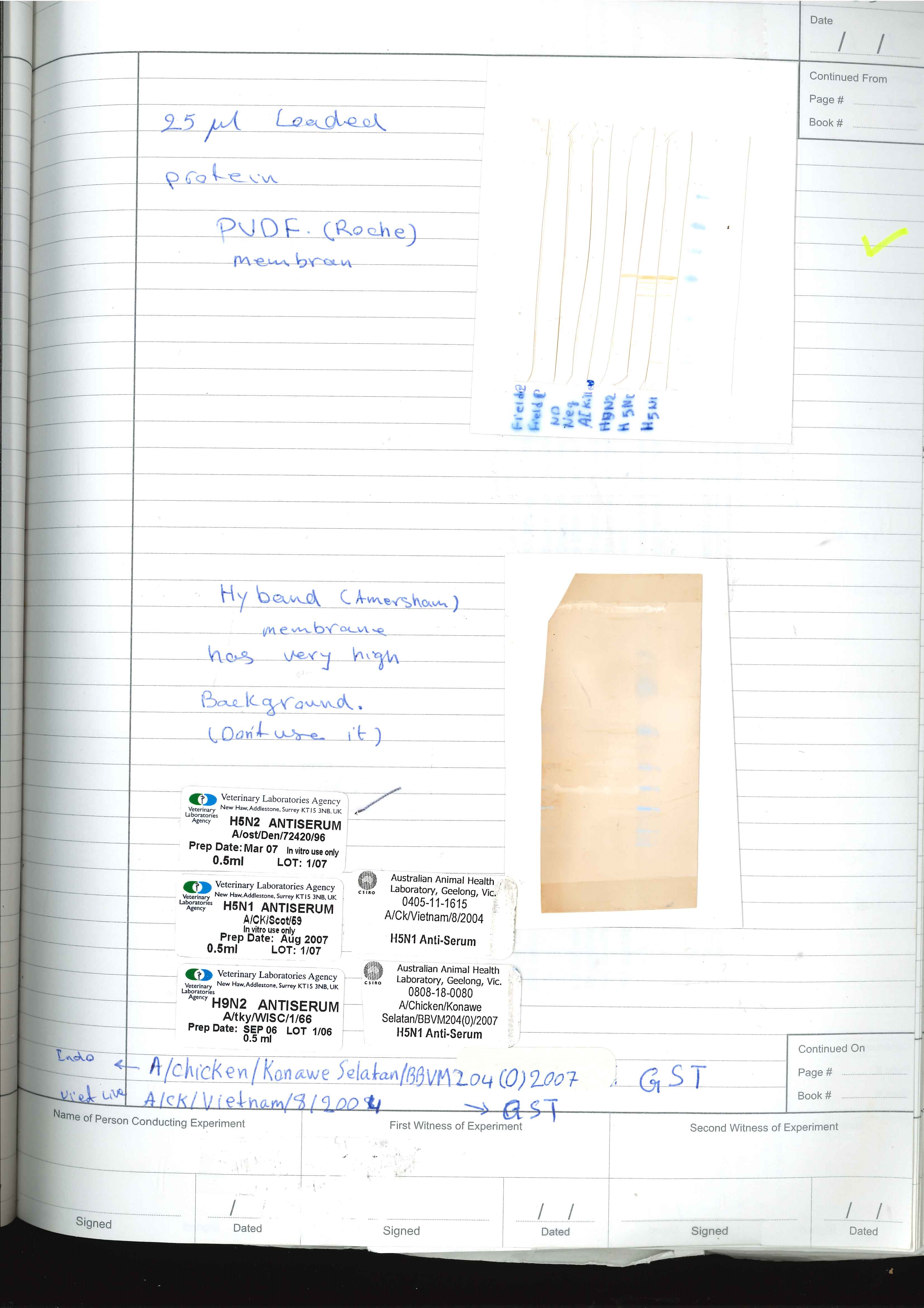

Supplement: S3 File — High-resolution image files of raw data. (ZIP) [file pone.0250485.s003.zip › P 39.jpg]

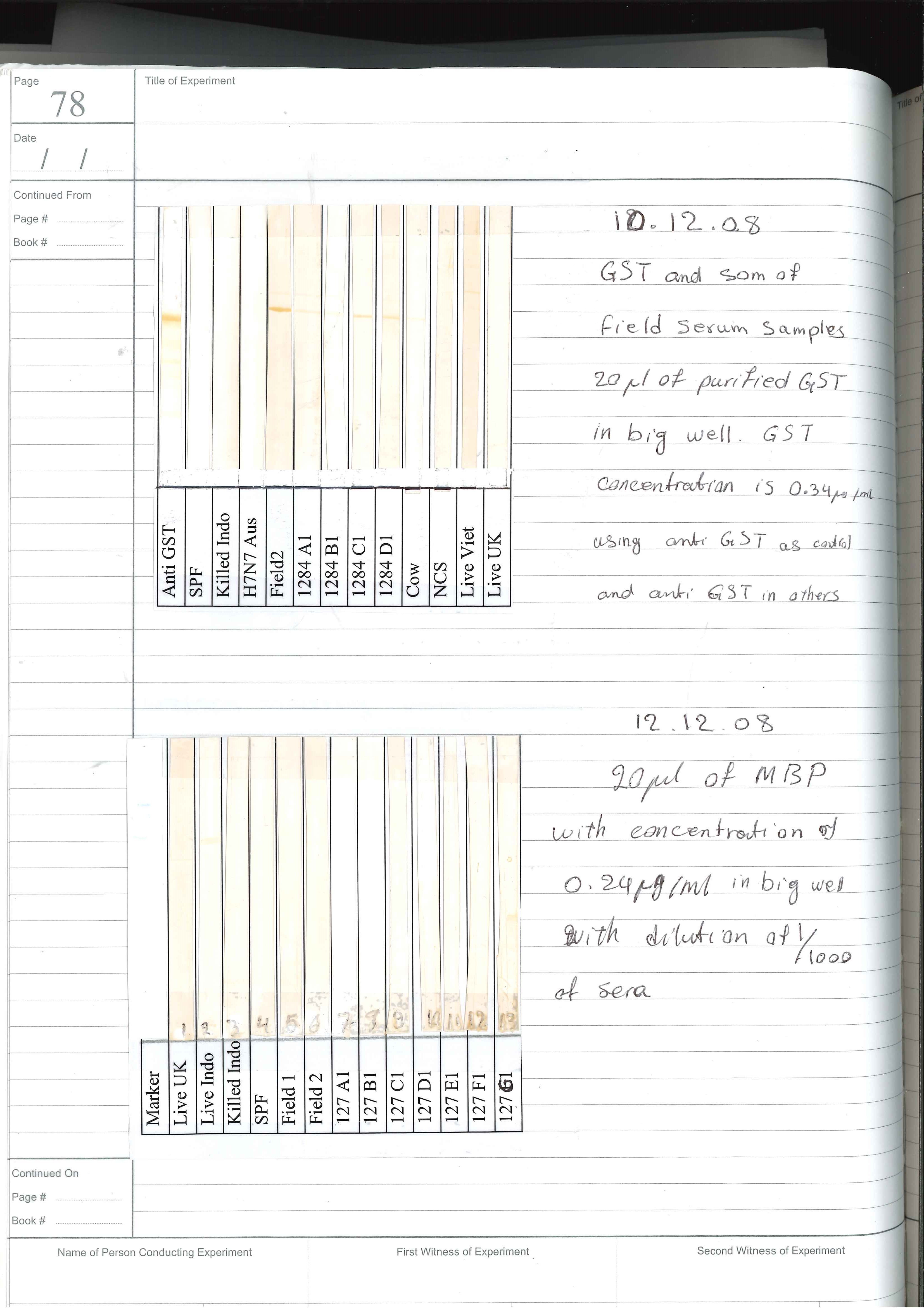

Supplement: S3 File — High-resolution image files of raw data. (ZIP) [file pone.0250485.s003.zip › P 78.jpg]

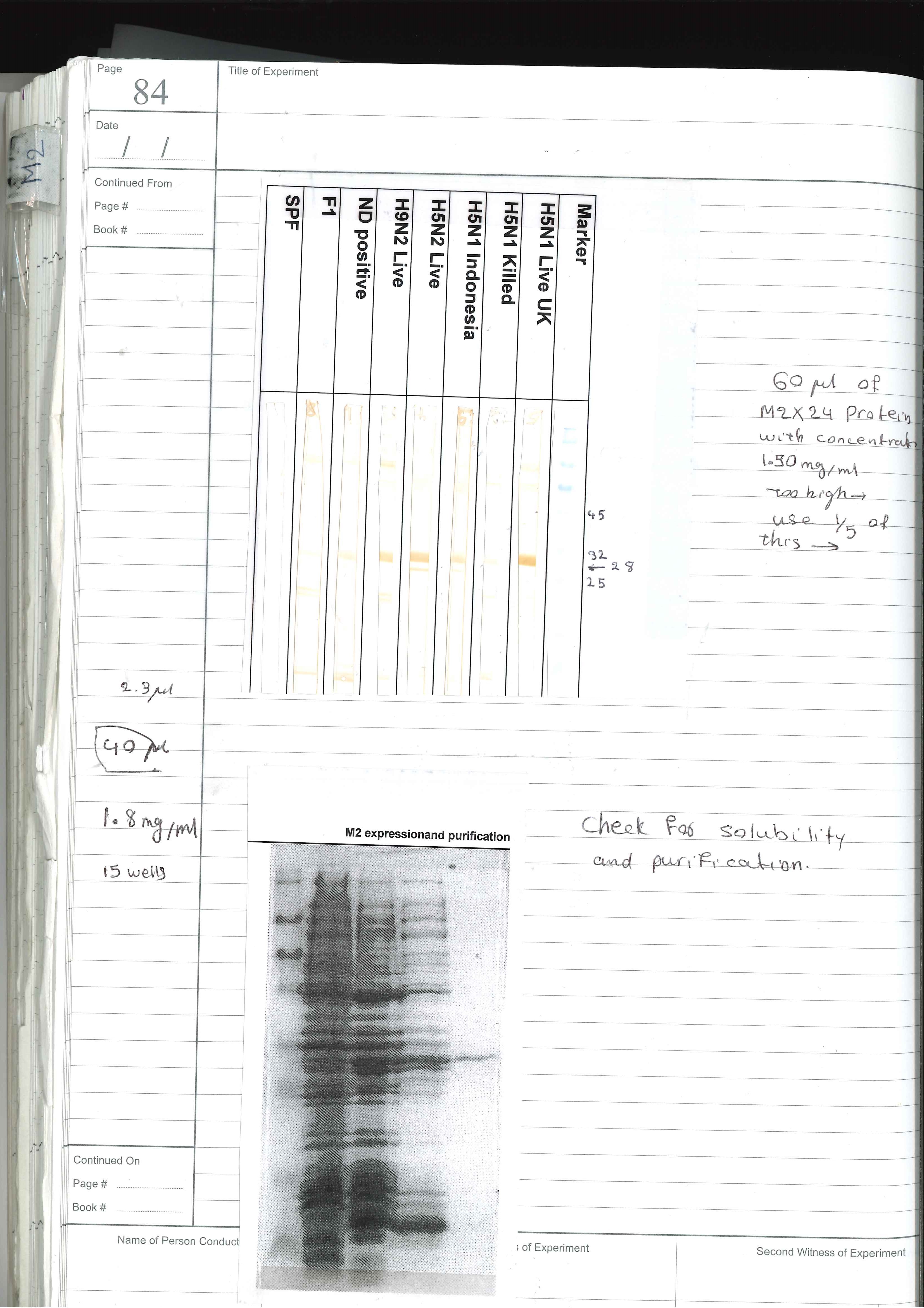

Supplement: S3 File — High-resolution image files of raw data. (ZIP) [file pone.0250485.s003.zip › P 84.jpg]

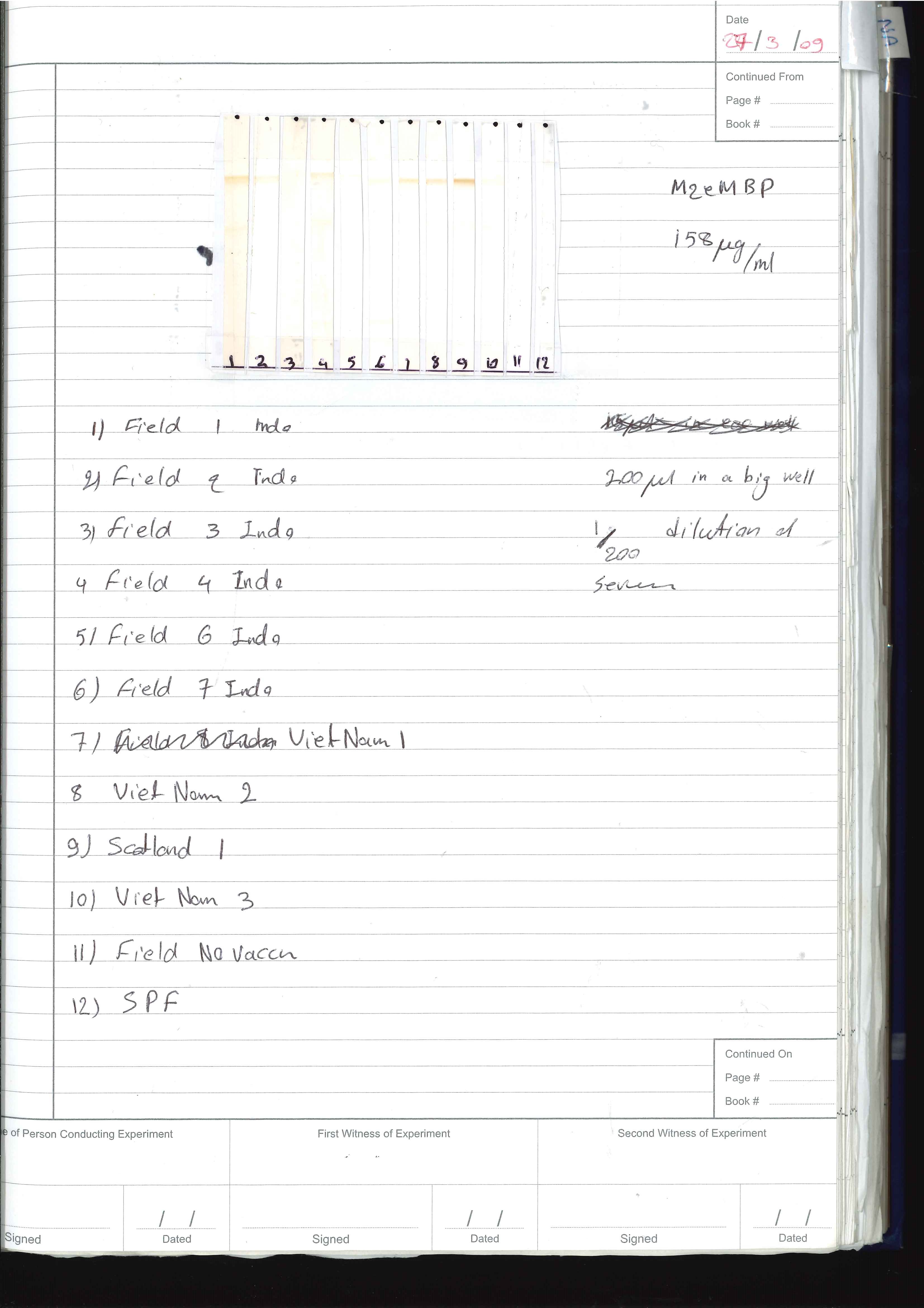

Supplement: S3 File — High-resolution image files of raw data. (ZIP) [file pone.0250485.s003.zip › P 93.jpg]

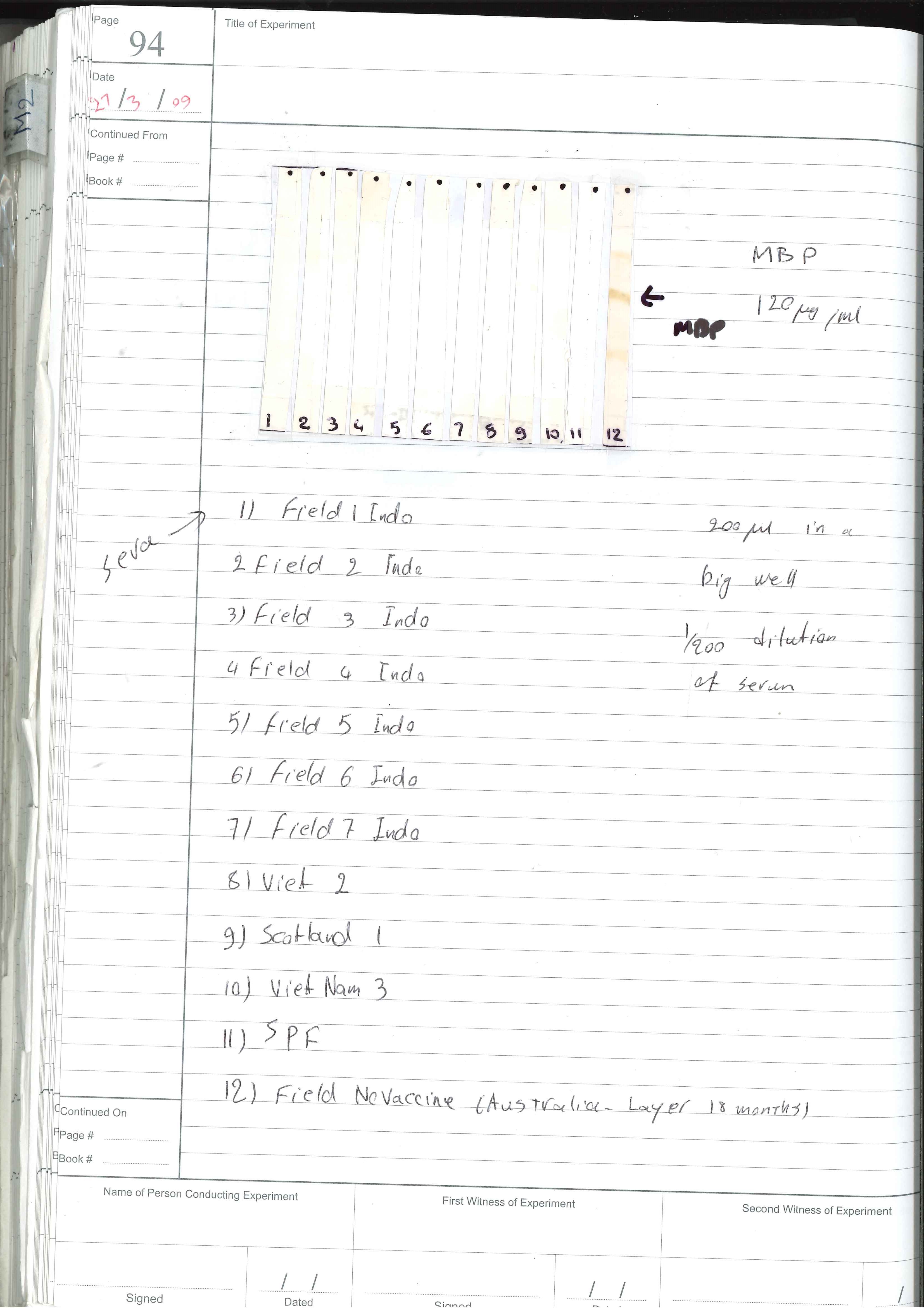

Supplement: S3 File — High-resolution image files of raw data. (ZIP) [file pone.0250485.s003.zip › P 94.jpg]

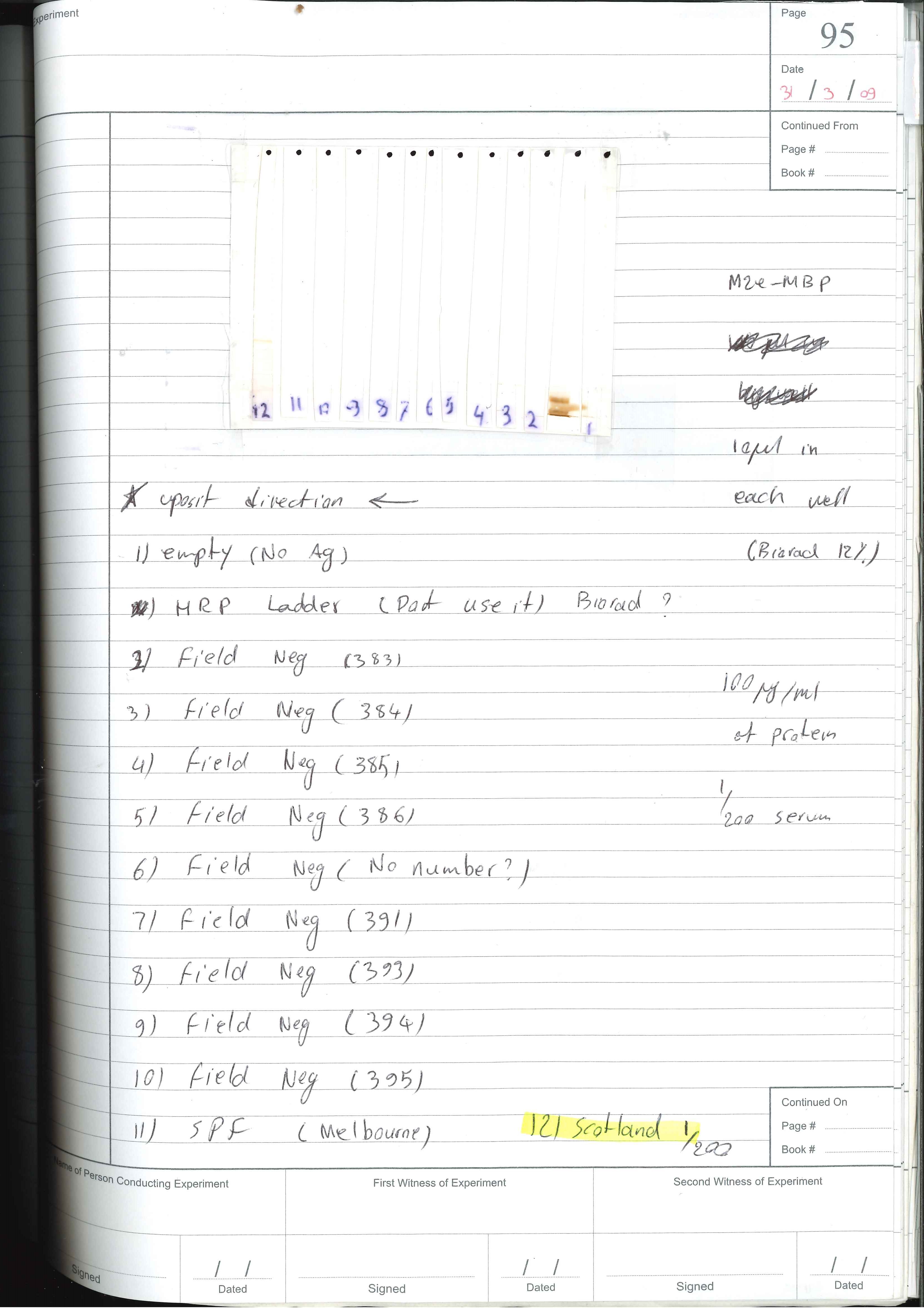

Supplement: S3 File — High-resolution image files of raw data. (ZIP) [file pone.0250485.s003.zip › P 95.jpg]

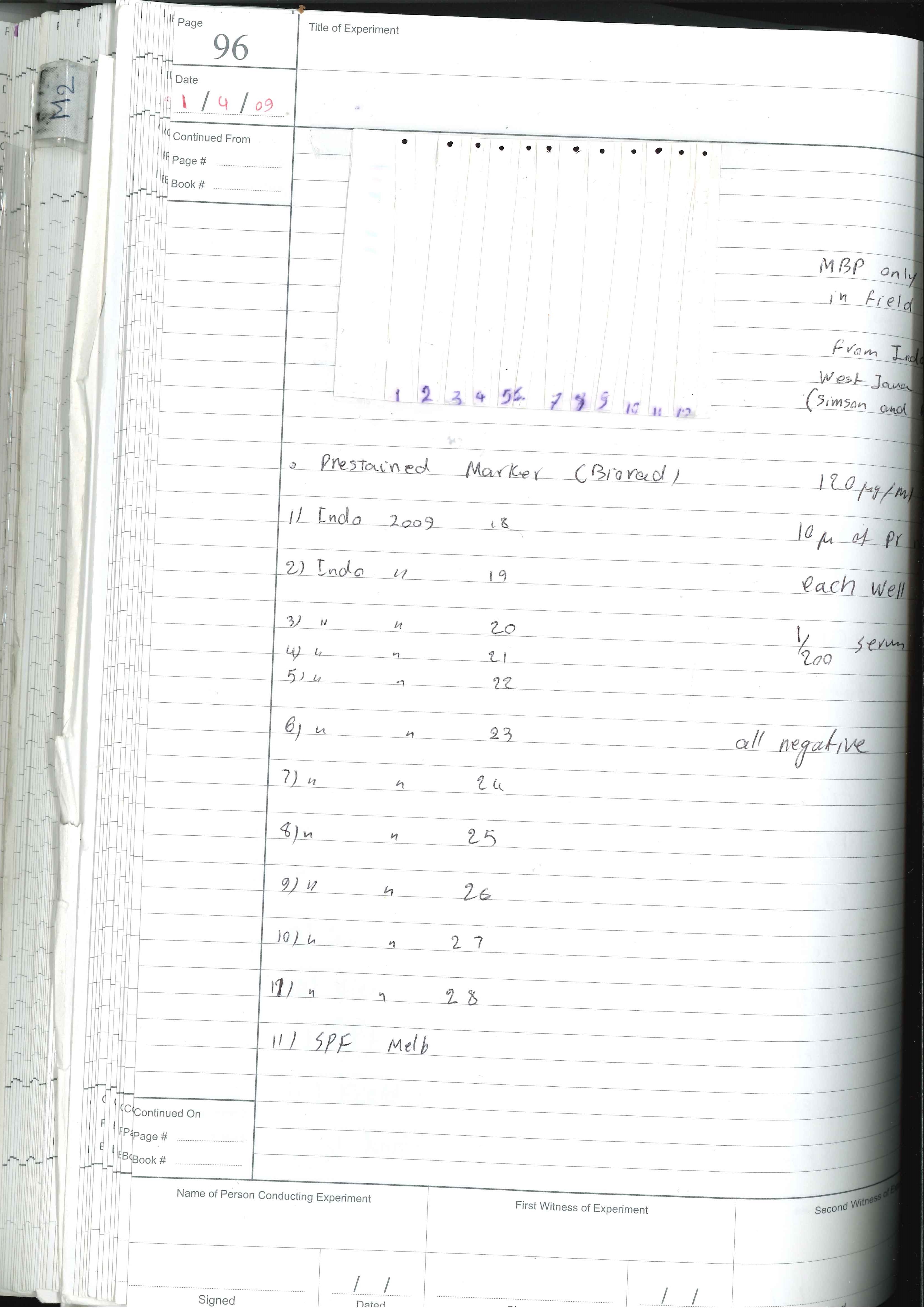

Supplement: S3 File — High-resolution image files of raw data. (ZIP) [file pone.0250485.s003.zip › P 96.jpg]

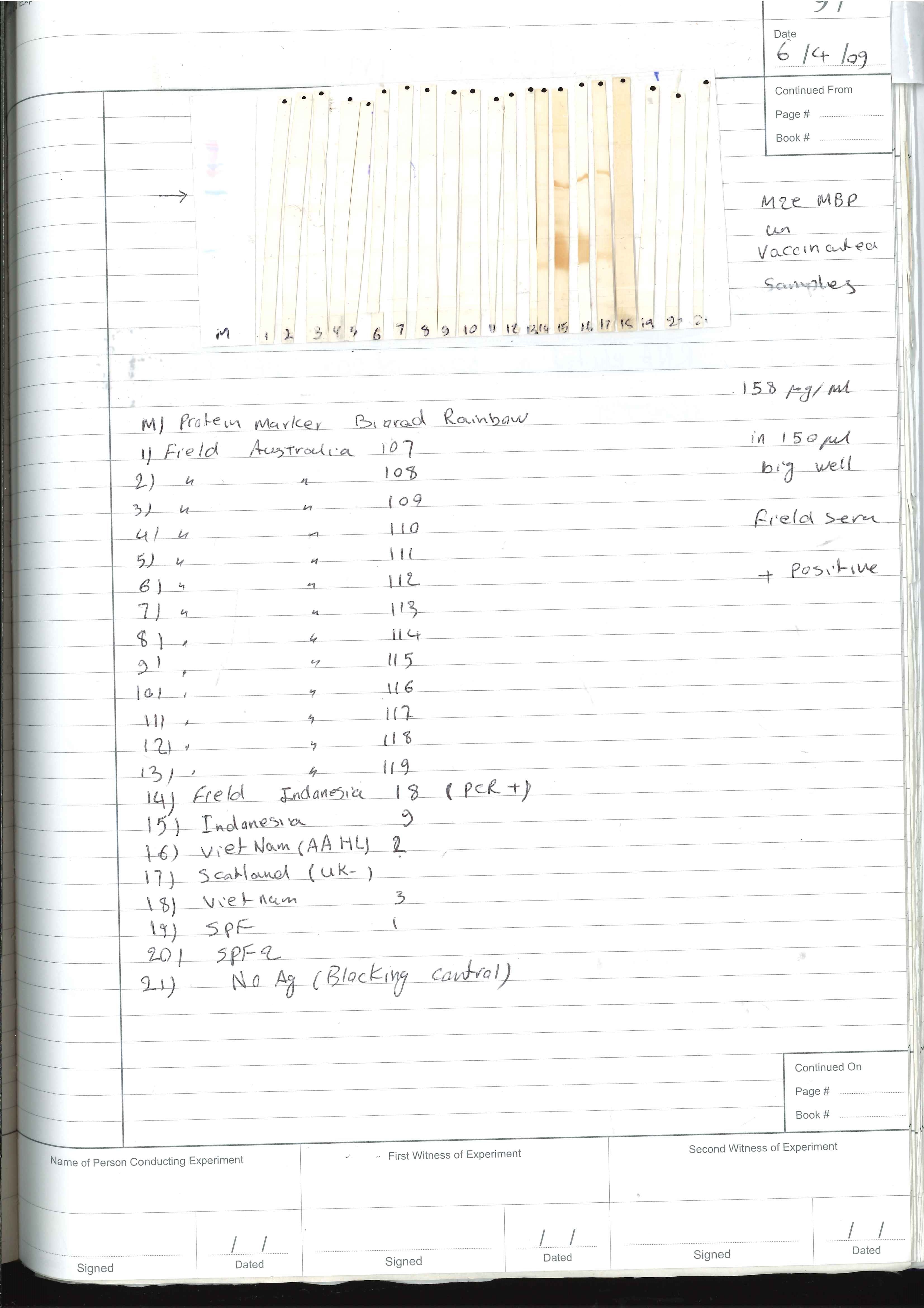

Supplement: S3 File — High-resolution image files of raw data. (ZIP) [file pone.0250485.s003.zip › P 97.jpg]

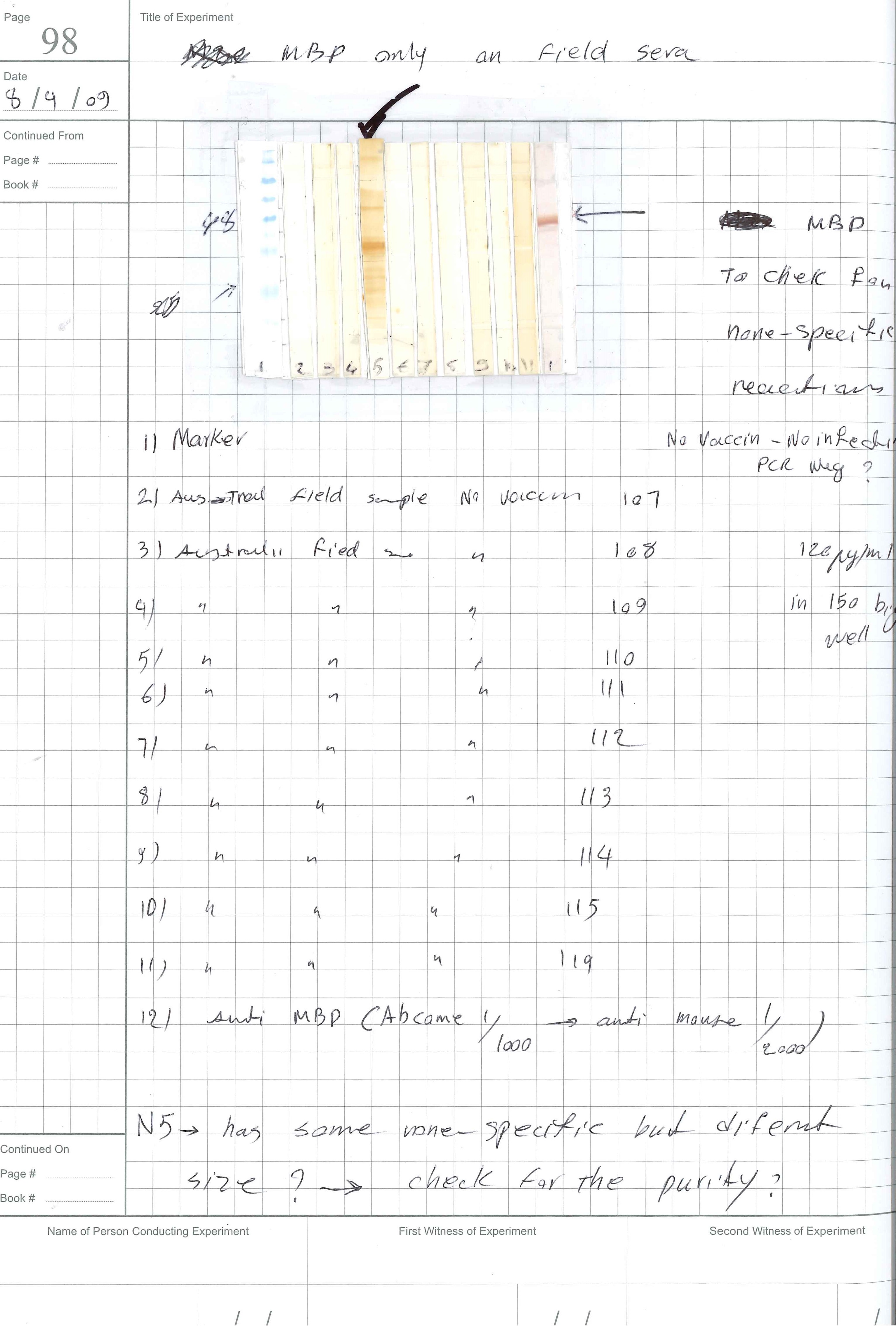

Supplement: S3 File — High-resolution image files of raw data. (ZIP) [file pone.0250485.s003.zip › P 98.jpg]
